# Supplementary material for: Characteristic Human Individual Puffing Profiles Can Generate More TNCO than ISO and Health Canada Regimes on Smoking Machine When the Same Brand Is Smoked
Source: Int J Environ Res Public Health. 2020 May 6;17(9):3225. doi: 10.3390/ijerph17093225 (PMC7246490; doi:10.3390/ijerph17093225)
Supplement: Supplementary file 1 [file ijerph-17-03225-s001.zip › Supp file Figure S1.docx]

Supplementary file B

Figure S1: Smoke escaping from the CReSSmicro™ device.


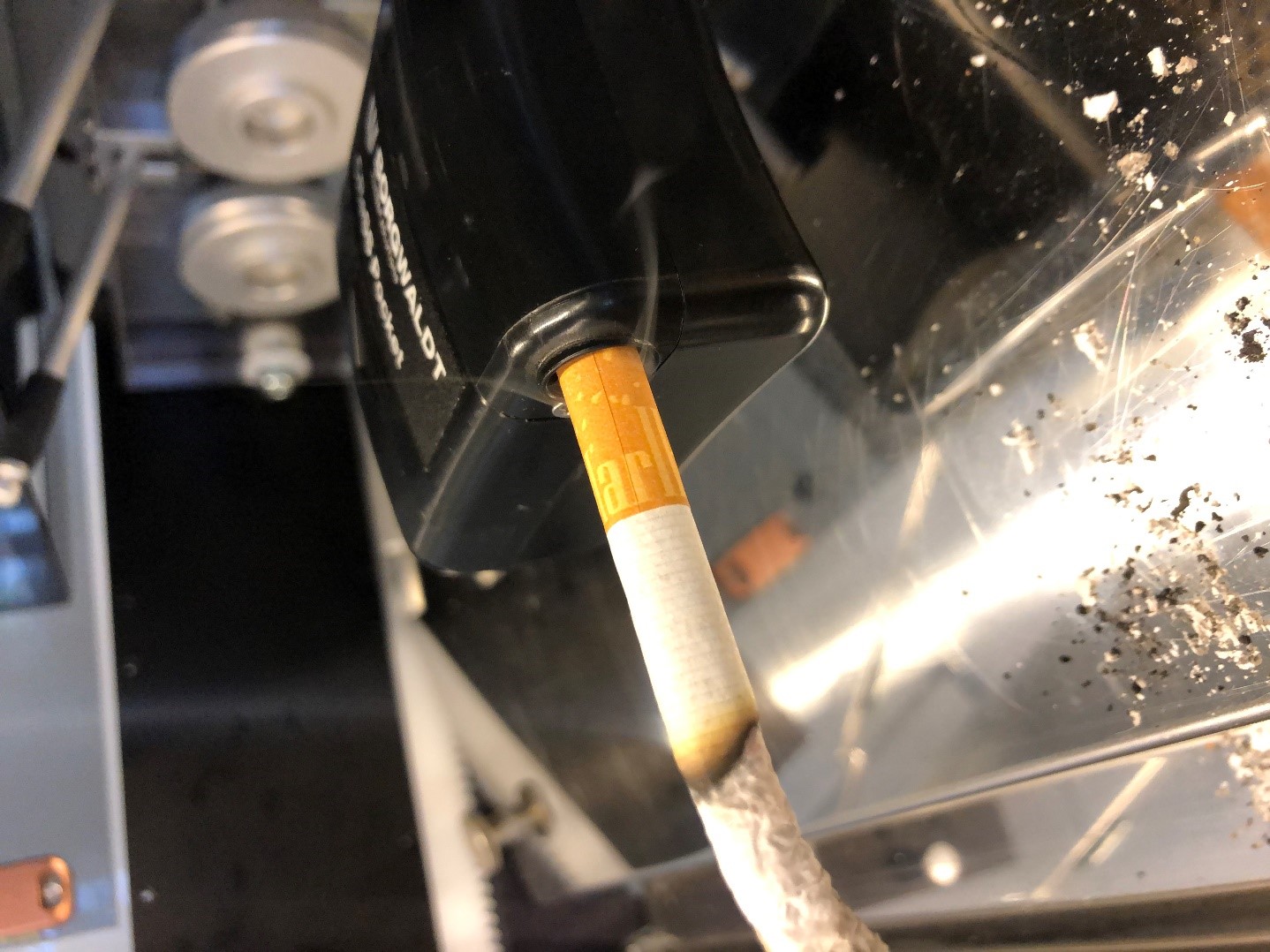


Figure S1: Smoke is escaping (arrow) during the smoking with a smoking machine. The cigarette was loosely inserted in the CReSSmicro™ device.
