# Supplementary material for: Characteristic Human Individual Puffing Profiles Can Generate More TNCO than ISO and Health Canada Regimes on Smoking Machine When the Same Brand Is Smoked
Source: Int J Environ Res Public Health. 2020 May 6;17(9):3225. doi: 10.3390/ijerph17093225 (PMC7246490; doi:10.3390/ijerph17093225)
Supplement: Supplementary file 1 [file ijerph-17-03225-s001.zip › Supp file Table S1.docx]

Supplementary file A

Table S1: Literature reporting puffing parameters in chronological order since 2000.

| Table S1: Literature reporting puffing parameters in chronological order since 2000 | | | | | | | | | | | | | | | | | | | | | | | | | |
| --- | --- | --- | --- | --- | --- | --- | --- | --- | --- | --- | --- | --- | --- | --- | --- | --- | --- | --- | --- | --- | --- | --- | --- | --- | --- |
| **Author** | **Year** | **Sort** | **n** | **Gender** | **age** | **SD** | **No of cig/day** | **SD** | **Puff volume (ml)** | **SD** | **Puff duration (sec)** | **SD** | **Puff flow** | **SD** | **Puff interval (sec)** | **SD** | **Puff count** | **SD** | **Total volume (ml/cig)** | **SD** | **Total volume/day** | **Fagerström index** | **SD** | **Device** | **Comments** |
| Buchhalter *et al* [1] | 2000 | own brand (9,5 mg tar, 0.73 mg nicotine) | 10 | ♂/♀ | 23.2 | 3.4 | 15.3 | 4.1 | 38.4 | 11.7 | 1.2 | 0.4 | *32.0* |  | 35.0 | 17.9 | 10.3 | 2.1 | *395.5* |  | *6.1* | 4.0 | 1.2 | CRSSPT |  |
| Djordjevic *et al* [2] | 2000 | own brand (8.5 mg tar, 0.7 mg nicotine) | 56 | ♂/♀ | 34.1 | CI 31.3-37.2 | 14.9 | CI 13.1-16.9 | 48.6 | CI 45.2 - 25.3 | 1.5 | CI 1.4-1.7 | *32.4* |  | 21.3 | CI 19-23.8 | 12.7 | CI 11.8-13.6 | 615.0 | CI 566-668 | 9.5 | 3.3 | CI 2.3-4.8 | Tobacco Smoke Inhalation Testing System |  |
|  |  | own brand (15.4 mg tar, 1.11 mg nicotine) | 77 | ♂/♀ | 34.7 | CI 32.5-37.1 | 15.9 | CI 14.3-17.8 | 44.1 | CI 40.8-46.8 | 1.5 | CI 1.4-1.6 | *29.4* |  | 18.5 | CI 16.5-20.6 | 12.1 | CI 11.3-12.9 | 523.0 | CI 487-561 | 8.2 | 3.8 | CI 2.7-5.3 | Tobacco Smoke Inhalation Testing System |  |
| Breland *et al* [3] | 2002 | own brand (10,2 mg tar, 0.8 mg nicotine) | 20 | ♂/♀ | 21.6 | SEM 1.1 | 18.9 | SEM 0.7 | 49.8 | SEM 3.3 | 1.7 | SEM 0.1 | *29.3* |  |  |  | 8 |  | *398.4* |  | *7.5* | 5.6 | SEM 0.3 | Tobacco Smoke Inhalation Testing System | Fixed puff count |
| Breland *et al* [4] | 2002 | own brand (9,4 mg tar, 0.73 mg nicotine) | 18 | ♂/♀ | 25.8 | 6.0 | 20.7 | 4.7 | 56.5 | 11.2 | 1.8 | 0.1 | *31.4* |  | 33.9 | 23.6 | 8 |  | *452.0* |  | *9.4* | 5.3 | 1.6 | Tobacco Smoke Inhalation Testing System | Fixed puff count |
| Dixon *et al* [5] | 2003 | test cigarette (9.5 mg tar, 0.77 mg nicotine) | 13 |  |  |  |  |  | 55.4 | 12.4 | 1.8 | 0.8 | *31.7* |  | 20.9 | 4.4 | 16.0 | 2.6 | 877.0 | 197.0 |  |  |  | Orifice-type holder/flowmeter (design of Creighton *et al*. 1978) |  |
|  |  | test cigarette (8.8 mg tar, 0.48 mg nicotine) | 13 |  |  |  |  |  | 56.7 | 11.6 | 1.8 | 0.8 | *31.5* |  | 20.8 | 3.6 | 16.1 | 3.0 | 890.0 | 152.0 |  |  |  | Orifice-type holder/flowmeter (design of Creighton *et al*. 1978) |  |
|  |  | test cigarette (8.7 mg tar, 0.81 mg nicotine) | 13 |  |  |  |  |  | 53.6 | 13.3 | 1.7 | 0.8 | *31.7* |  | 21.4 | 5.3 | 14.4 | 2.3 | 755.0 | 166.0 |  |  |  | Orifice-type holder/flowmeter (design of Creighton *et al*. 1978) |  |
|  |  | test cigarette 8.1mg tar, 0.10 mg nicotine) | 13 |  |  |  |  |  | 55.6 | 11.5 | 1.8 | 0.7 | *31.4* |  | 21.1 | 4.5 | 14.4 | 2.3 | 766.0 | 141.0 |  |  |  | Orifice-type holder/flowmeter (design of Creighton *et al*. 1978) |  |
|  |  | test cigarette (7.7 mg tar, 0.22 mg nicotine) | 13 |  |  |  |  |  | 55.8 | 13.0 | 1.8 | 0.8 | *30.3* |  | 20.0 | 3.7 | 15.6 | 3.3 | 870.0 | 136.0 |  |  |  | Orifice-type holder/flowmeter (design of Creighton *et al*. 1978) |  |
| Lee *et al* [6] | 2003 | own brand (16.7 mg tar, 1.2 mg nicotine) | 7 | ♂/♀ | 37.0 | 25-44 | 25.0 | 10-38 | 30.8 | SE 3.5 | 0.9 | SE 0.1 | 44.6 | SE 3.4 | 19.9 | SE 3.5 | *9.3* |  | *284.9* |  | *7.1* | 6.8 | 4-10 | CRSSPT | 8, 8, 11, 10 puffs |
|  |  | own brand (12.8 mg tar, 1.0 mg nicotine) | 10 | ♂/♀ | 24.5 | 20-37 | 25.0 | 17-40 | 45.1 | SE 4.9 | 1.2 | SE 0.1 | 54.4 | SE 4.8 | 23.2 | SE 3.0 | 10.0 |  | *451.0* |  | *11.3* | 6.0 | 3-9 | CRSSPT |  |
| Aung *et al* [7] | 2004 | own brand | 56 | ♂/♀ | 15.4 | 1.3 | 19.6 | 8.93 | 38.1 | 14.5 | 1.1 | 0.4 | 52.0 | 19.3 | 15.5 | 8.6 | 14.0 | 3.9 | *534.0* |  | *10.5* | 7.4 | 1.4 | CRSSPT | Current non-marijuana-smoking teens |
| Dixon^$^ | 2004 | 10 mg tar, 1 mg nicotine | 100 | ♂/♀ |  |  |  |  | 46.2 |  | 1.9 |  | *24.2* |  | 24.7 |  | 12.6 |  | 558.0 |  |  |  |  |  |  |
|  |  | 4,8 mg tar, 0.5 mg nicotine | 100 | ♂/♀ |  |  |  |  | 58.6 |  | 1.9 |  | *31.7* |  | 21.7 |  | 16.6 |  | 939.0 |  |  |  |  |  |  |
|  |  | 2,8 mg tar, 0.3 mg nicotine | 100 | ♂/♀ |  |  |  |  | 57.6 |  | 1.9 |  | *30.6* |  | 19.7 |  | 15.0 |  | 844.0 |  |  |  |  |  |  |
|  |  | 1,2 mg tar, 0.1 mg nicotine | 100 | ♂/♀ |  |  |  |  | 63.4 |  | 1.9 |  | *33.2* |  | 18.7 |  | 18.8 |  | 1135.0 |  |  |  |  |  |  |
| Dixon^$^ | 2004 | 5,6 mg tar, 0.5 mg nicotine | 50 | ♂/♀ |  |  |  |  | 52.8 |  | 1.7 |  | *30.3* |  |  |  | 12.5 |  | 644.0 |  |  |  |  |  |  |
|  |  | 1 mg tar, 0.1 mg nicotine | 50 | ♂/♀ |  |  |  |  | 63.2 |  | 1.7 |  | *36.3* |  |  |  | 15.1 |  | 901.0 |  |  |  |  |  |  |
| Hughes *et al* [8] | 2004 | own brand (Light, ultralight, regular) | 34 | ♂/♀ | 48.0 |  | 30.0 | SEM 2 | 50.0 | SEM 2 |  |  |  |  |  |  | 12.7 | SEM 0.7 | 612.0 | SEM 34 | *18.4* | 6.4 | 2.0 | CRSSPT |  |
|  |  | Omni light (12 mg tar, 0.8 mg nicotine) or Omni Full flavor King/100's (15 mg tar, 1.0 mg nicotine) | 34 | ♂/♀ | 48.0 |  | 31.0 | SEM 2 | 49.0 | SEM 2 |  |  |  |  |  |  | 11.6 | SEM 0.5 | 547.0 | SEM 25 | *17.0* | 6.4 | 2.0 | CRSSPT |  |
| Lee *et al* [9] | 2004 | own brand (14.5 mg tar, 1.1 mg nicotine) | 10 | ♂/♀ | 30.3 | 19-46 | 21.3 | 13-30 | 60.1 | 4.0 | *1.1* |  | 57.1 | 3.5 |  |  | 11.5 | 0.7 | 691.2 |  | *14.7* | 5.4 | 3-9 | CRSSPT |  |
| Strasser *et al* [10] | 2004 | own brand (mean 0.93mg nicotine) | 113 | ♂/♀ | 45.8 | 10.7 | 21.2 | 7.8 | 54.8 | 16.9 | 1.6 | 0.5 | 34.3 | 8.2 | 24.8 | 11.9 | 12.7 | 4.0 | 675.7 | 244.7 | *14.3* | 5.5 | 2.1 | CRSSPT |  |
| Wood *et al* [11] | 2004 | own brand | 24 | ♀ | 16.3 | 1.5 | 15.0 | 1-25 | 38.5 | 12.2 | 1.1 | 0.3 | *35.0* |  | 24.1 | 9.9 | 12.7 | 6.2 | 504.4 | 312.1 | 7.6 | 4.2 | 1.6 | CRSSPT |  |
|  |  | own brand | 25 | ♂ | 16.4 | 1.4 | 15.0 | 1-25 | 45.8 | 14.6 | 1.2 | 0.3 | *38.2* |  | 19.1 | 10.9 | 15.6 | 6.1 | 729.0 | 393.5 | 10.9 | 4.6 | 1.7 | CRSSPT |  |
| Zimmerman *et al* [12] | 2004 | own brand | 92 | ♂/♀ | 15.2 | 1.5 |  |  | 38.2 | 12.5 | 1.1 | 0.4 | 51.1 | 16.0 | 16.8 | 10.9 |  |  |  |  |  |  |  | CRSSPT | volunteers cessation trial NIDA |
| Hammond *et al* [13] | 2005 | own brand (10-14 mg tar) | 58 | ♂/♀ | 37.1 | 11.1 | 19.3 | 8.0 | 54.2 | 10.3 | 1.5 | 0.2 | 38.1 | 5.6 | 33.5 | 17.6 | 11.3 | 3.4 | 612.0 | 195.7 | *11.8* |  |  | CRSSPT | trial 1 |
|  |  | own brand (10-14 mg tar) | 58 | ♂/♀ | 37.1 | 11.1 | 19.3 | 8.0 | 52.3 | 12.4 | 1.4 | 0.3 | 37.9 | 6.1 | 35.6 | 18.4 | 11.1 | 3.3 | 580.5 | 206.9 | *11.2* |  |  | CRSSPT | trial 2 |
|  |  | Matinee Extra Mild (4 mg tar, 0.8 mg nicotine) | 24 | ♂/♀ | 37.1 | 11.1 | 19.3 | 8.0 | 58.3 | 16.1 |  |  |  |  |  |  | *13.4* |  | 779.2 | 331.0 | *15.0* |  |  | CRSSPT | trial 3 |
| Hitsman *et al* [14] | 2005 | own brand (mean 0.8 mg nicotine) | 8 | ♂/♀ | 35.5 | 16.9 | 18.8 | 2.3 |  |  | 2.4 | 0.7 |  |  | 36.8 | 17.2 | 10.8 | 3.6 |  |  |  | 5.6 | 1.7 | Videotaping + handtiming |  |
| Strasser *et al* [15] | 2005 | Marlboro Light 100 HP (10 mg tar, 0.8 mg nicotine) | 12 | ♂/♀ | 21.6 | 4.5 | 17.5 | 12.5 | 63.4 | SE 8.4 | 1.6 | SE 0.2 | 42.5 | SE 3.2 | 45 |  | 8 |  | *507.2* |  | *8.9* |  |  | CReSS | Study 1, fixed puff count and IPI, unblocked |
|  |  | Carlton 100 HP (1 mg tar, 0.1 mg nicotine) | 12 | ♂/♀ | 21.6 | 4.5 | 18.0 | 12.5 | 85.1 | SE 10.9 | 2.0 | SE 0.3 | 44.5 | SE 2.8 | 45 |  | 8 |  | *680.8* |  | *12.3* |  |  | CReSS | Study 1, fixed puff count and IPI, unblocked |
|  |  | Carlton 100 HP (1 mg tar, 0.1 mg nicotine) | 12 | ♂ | 41.6 | 15.3 | 19.4 | 9.5 | 83.9 |  | 1.9 |  | *62.2* |  | 45 |  | 8 |  | *671.5* |  | *13.0* | 4.0 | 2.6 | CReSS | Study 2, fixed puff count and IPI, unblocked, calculated mean (n=3) |
| Tidey *et al* [16] | 2005 | own brand (mean 1.1 mg nicotine) | 20 | ♂/♀ | 51.0 | 12.0 | 29.0 | 11 | 47.5 | 10.4 | 1.3 | 0.2 | *36.5* |  | 42.0 | 21.5 | 8.9 | 2.3 | 429.0 | 159.0 | *12.4* | 7.4 | 1.3 | CReSS |  |
| Breland *et al* [17] | 2006 | own brand (9.89 mg tar, 0.74 mg nicotine) | 35 | ♂/♀ | 22.4 | CI 21.19-23.55 | 21.0 | CI 3.46-5.55 | 51.0 | CI 45.70-56.24 | 1.4 | CI 1.24-1.52 | *36.9* |  | 30.7 | CI 26.48-34.99 | 10.0 | CI 8.85-11.21 | *511.2* |  | *10.8* |  |  | CReSS |  |
| Franken *et al* [18] | 2006 | own brand | 66 | ♂/♀ | 15.1 | 1.3 | 19.0 | 8.4 | 36.0 | 13.5 | 1.1 | 0.4 | 35.6 | 10.6 | 18.2 | 10.6 | 5 |  | *180.0* |  | *3420.0* | 7.2 | 1.3 | CReSS | Fixed puff count |
| Hammond *et al* [19] | 2006 | own brand (9-15 mg tar) | 51 | ♂/♀ | 37.1 |  | >5 |  | 53.3 | 11.9 | 1.4 | 0.3 | 38.6 | 5.9 | 33.2 | 15.8 | 11.5 | 3.6 | 602.6 | 195.8 | *3013.0* |  |  |  | Same data as Hammond et al [13] |
|  |  | Matinee Extra Mild (4 mg tar, 0.4 mg nicotine) | 21 | ♂/♀ | 37.1 |  | >5 |  | 60.7 | 15.6 | 1.6 | 0.4 | 40.8 | 8.4 | 30.5 | 15.4 | 13.4 | 5.3 | 802.4 | 346.2 | *4012.0* |  |  |  | Same data as Hammond et al [13] |
| Kassel *et al* [20] | 2007 | Research cigarette (15.9 mg tar, 1.14 mg nicotine) | 16 | ♂/♀ | 17.5 | 1.1 | 3.4 | 1.2 | 45.4 | 22.2 | 1.1 | 0.4 | 57.2 | 18.9 | 24.2 | 12.9 | 15.1 | 6.3 | 713.1 | 461.8 | *2.4* | 2.2 | 1.4 | CReSS |  |
|  |  | Research cigarette (17.9 mg tar, 0.06 mg nicotine) | 19 | ♂/♀ | 17.5 | 1.1 | 3.8 | 2 | 40.1 | 18.7 | 1.0 | 0.4 | 53.4 | 19.0 | 23.0 | 13.2 | 23.2 | 8.9 | 988.8 | 547.2 | *3.8* | 2.1 | 1.4 | CReSS |  |
| Melikian *et al* [21] | 2007 | own brand (10.1 mg tar, 1.92 mg nicotine) | 129 | ♀ | 33.1 | CI 31.2-35 | 15.9 | CI 14.7-17.1 | 37.6 |  | 1.3 |  | *28.3* |  | 20.4 |  | 13.5 |  | 507.8 |  | *8.1* | 4.1 | CI 3.65-4.62 | puff analyzer [22] |  |
|  |  | own brand (12.4 mg tar, 2.2 mg nicotine) | 128 | ♂ | 35.0 | CI 33.3-36.9 | 16.8 | CI 15.5-18.2 | 45.8 |  | 1.5 |  | *30.9* |  | 20.7 |  | 12.0 |  | 553.0 |  | *9.3* | 4.6 | CI 4.12-5.06 | puff analyzer [22] |  |
| Strasser *et al* [23] | 2007 | own brand (mean 12,9 mg tar, 0.94 mg nicotine) | 50 | ♂/♀ | 44.5 | 12.1 | 21.3 | 8.1 | 60.5 | CI 55.2-65.7 | 1.8 | CI 1.6-1.9 | 35.5 | CI 22.3-37.8 | 21.6 | CI 19.1-24.1 | 14.3 | CI 12.8-15.7 | 832.0 | CI 737-926 | *17.7* | 5.5 | 1.9 | CReSS |  |
|  |  | Quest (10 mg tar, 0.6 mg nicotine) | 50 | ♂/♀ | 44.5 | 12.1 | 21.3 | 8.1 | 58.1 | CI 53.3-62.8 | 1.8 | CI 1.6-1.9 | 34.5 | CI 32.0-37.0 | 21.6 | CI 19.0-24.2 | 9.8 | CI 9-10.8 | 540.3 | CI 500-580 | *11.5* | 5.5 | 1.9 | CReSS |  |
|  |  | Quest (10 mg tar, 0.3 mg nicotine) | 50 | ♂/♀ | 44.5 | 12.1 | 21.3 | 8.1 | 55.9 | CI 51.0-60.8 | 1.7 | CI 1.5-1.8 | 34.4 | CI 31.9-37.0 | 19.6 | CI 16.9-22.3 | 9.9 | CI 8.9-10.9 | 518.1 | CI 478-558 | *11.0* | 5.5 | 1.9 | CReSS |  |
|  |  | Quest (10 mg tar, 0.05 mg nicotine) | 50 | ♂/♀ | 44.5 | 12.1 | 21.3 | 8.1 | 59.4 | CI 54.6-64.3 | 1.8 | CI 1.6-1.9 | 35.1 | CI 32.5-37.8 | 18.6 | CI 15.7-21.6 | 10.0 | CI 9.1-10.9 | 570.5 | CI 527-614 | *12.2* | 5.5 | 1.9 | CReSS |  |
| Strasser *et al* [24] | 2007 | own brand | 119 | ♂/♀ | 45.8 | 10.8 | 21.2 | 8.1 | 55.1 | 16.9 |  |  |  |  |  |  | 12.7 | 4.0 | 669.6 | 243.1 | *14.2* | 5.5 | 2.1 | CReSS |  |
| Attwood *et al* [25] | 2008 | own brand | 55 | ♂/♀ | 22.0 | 4.0 | 11.0 | 5 | 49.0 | 12.0 | 1.6 | 0.4 | *31.6* |  | 14.0 | 5.0 | 17.0 | 6.0 | *833.0* |  | *9.2* | 4.0 | 2.0 | CReSS |  |
| Collins *et al* [26] | 2008 | own brand | 67 | ♂/♀ | 15.3 | 1.3 | >10 |  | 38.7 |  | 1.1 |  | 48.1 |  |  |  | 6 |  | *232.1* |  | *2.3* | >5 |  | CReSS | Fixed puff count |
| Rees *et al* [27] | 2008 | Marlboro Light (11 mg tar, 0.8 mg nicotine) | 17 | ♂/♀ | 27.7 | CI 24.3-31.0 | 15.7 | CI 12.0-19.3 | 50.7 | 19.6 | 1.4 | 0.5 | *37.6* |  | 32.1 | 11.9 | 11.4 | 3.0 | *578.0* |  | *9.1* | 3.9 | CI 2.5-5.4 | CReSS | Supervised - Tampa group |
|  |  | Marlboro Light (11 mg tar, 0.8 mg nicotine) | 17 | ♂/♀ | 27.7 | CI 24.3-31.0 | 15.7 | CI 12.0-19.3 | 51.2 | 17.3 | 1.4 | 0.5 | *35.8* |  | 37.3 | 22.1 | 11.9 | 4.0 | *609.3* |  | *9.6* | 3.9 | CI 2.5-5.4 | CReSS | Unsupervised - Tampa group |
|  |  | Marlboro Light (11 mg tar, 0.8 mg nicotine) | 15 | ♂/♀ | 27.8 | CI 35.5-32.1 | 15.9 | CI 9.5-22.2 | 47.4 | 16.9 | 1.3 | 0.4 | *36.7* |  | 24.8 | 11.5 | 13.1 | 4.8 | *620.9* |  | *9.9* | 4.4 | CI 3.0-5.8 | CReSS | Supervised - Salt Lake City group |
|  |  | Marlboro Light (11 mg tar, 0.8 mg nicotine) | 15 | ♂/♀ | 27.8 | CI 35.5-32.1 | 15.9 | CI 9.5-22.2 | 41.0 | 18.0 | 1.3 | 0.5 | *32.3* |  | 26.8 | 10.4 | 11.9 | 4.7 | *487.9* |  | *7.8* | 4.4 | CI 3.0-5.8 | CReSS | Unsupervised - Salt Lake City group |
|  |  | Marlboro ultraLight (6 mg tar, 0.5 mg nicotine) | 17 | ♂/♀ | 27.7 | CI 24.3-31.0 | 15.7 | CI 12.0-19.3 | 51.4 | 19.0 | 1.3 | 0.4 | *38.9* |  | 33.0 | 14.9 | 11.3 | 2.6 | *580.8* |  | *9.1* | 3.9 | CI 2.5-5.4 | CReSS | Supervised - Tampa group |
|  |  | Marlboro ultraLight (6 mg tar, 0.5 mg nicotine) | 17 | ♂/♀ | 27.7 | CI 24.3-31.0 | 15.7 | CI 12.0-19.3 | 49.9 | 18.3 | 1.4 | 0.5 | *35.6* |  | 35.1 | 15.3 | 10.5 | 2.6 | *524.0* |  | *8.2* | 3.9 | CI 2.5-5.4 | CReSS | Unsupervised - Tampa group |
|  |  | Marlboro ultraLight (6 mg tar, 0.5 mg nicotine) | 15 | ♂/♀ | 27.8 | CI 35.5-32.1 | 15.9 | CI 9.5-22.2 | 50.1 | 15.6 | 1.4 | 0.4 | *36.3* |  | 21.8 | 10.4 | 13.9 | 5.6 | *696.4* |  | *11.1* | 4.4 | CI 3.0-5.8 | CReSS | Supervised - Salt Lake City group |
|  |  | Marlboro ultraLight (6 mg tar, 0.5 mg nicotine) | 15 | ♂/♀ | 27.8 | CI 35.5-32.1 | 15.9 | CI 9.5-22.2 | 52.2 | 15.7 | 1.5 | 0.5 | *34.8* |  | 24.7 | 12.3 | 12.1 | 4.6 | *631.6* |  | *10.0* | 4.4 | CI 3.0-5.8 | CReSS | Unsupervised - Salt Lake City group |
|  |  | Marlboro UltraSmooth (5 mg tar, 0.4 mg nicotine) | 17 | ♂/♀ | 27.7 | CI 24.3-31.0 | 15.7 | CI 12.0-19.3 | 54.2 | 19.4 | 1.4 | 0.4 | *39.0* |  | 28.4 | 8.4 | 10.2 | 2.9 | *552.8* |  | *8.7* | 3.9 | CI 2.5-5.4 | CReSS | Activated carbon in filter - Supervised - Tampa group |
|  |  | Marlboro UltraSmooth (5 mg tar, 0.4 mg nicotine) | 17 | ♂/♀ | 27.7 | CI 24.3-31.0 | 15.7 | CI 12.0-19.3 | 53.5 | 19.2 | 1.4 | 0.5 | *37.4* |  | 33.6 | 15.8 | 9.3 | 2.4 | *497.6* |  | *7.8* | 3.9 | CI 2.5-5.4 | CReSS | Activated carbon in filter - Unsupervised - Tampa group |
|  |  | Marlboro UltraSmooth (5 mg tar, 0.4 mg nicotine) | 15 | ♂/♀ | 27.8 | CI 35.5-32.1 | 15.9 | CI 9.5-22.2 | 56.7 | 15.2 | 1.5 | 0.4 | *38.6* |  | 23.9 | 9.2 | 12.5 | 5.0 | *708.8* |  | *11.3* | 4.4 | CI 3.0-5.8 | CReSS | Activated carbon in filter - Supervised - Salt Lake City group |
|  |  | Marlboro UltraSmooth (5 mg tar, 0.4 mg nicotine) | 15 | ♂/♀ | 27.8 | CI 35.5-32.1 | 15.9 | CI 9.5-22.2 | 54.6 | 19.4 | 1.5 | 0.5 | *35.9* |  | 24.6 | 12.2 | 11.7 | 5.2 | *638.8* |  | *10.2* | 4.4 | CI 3.0-5.8 | CReSS | Activated carbon in filter - Unsupervised - Salt Lake City group |
| Shahab *et al* [28] | 2008 | own brand | 131 | ♂/♀ | 31.4 | 10.4 | 13.4 | 5.7 | 54.2 | 14.0 | 1.5 | 0.5 | 37.7 | 7.8 | 25.8 | 10.3 | 13.6 | 4.5 | 712.0 | 232.0 | *9.5* | 2.3 | 1.4 | CReSS |  |
| Blank *et al* [29] | 2009 | own brand (mean 14.9 mg tar, 1.1 mg nicotine) | 30 | ♂/♀ | 32.3 | 11.0 | 19.6 | 4.7 | 44.4 | 13.6 | 1.7 | 0.6 | *26.1* |  | 18.2 | 7.6 | 9.4 | 3.0 | 411.4 | 170.7 | *8.1* | 6.0 | 2.0 | Portable CReSS |  |
|  |  | own brand (mean 14.9 mg tar, 1.1 mg nicotine) | 30 | ♂/♀ | 32.3 | 11.0 | 19.6 | 4.7 | 57.0 | 20.1 | 1.7 | 0.7 | *33.5* |  | 18.1 | 8.8 | 9.7 | 3.3 | 578.7 | 327.2 | *8.1* | 6.0 | 2.0 | Desktop CReSS |  |
|  |  | Merit ultra-light (5 mg tar, 0.5 mg nicotine) | 30 | ♂/♀ | 32.3 | 11.0 | 19.6 | 4.7 | 52.8 | 12.6 | 1.9 | 0.6 | *27.8* |  | 16.7 | 7.7 | 10.1 | 3.4 | 528.5 | 207.2 | *10.4* | 6.0 | 2.0 | Portable CReSS |  |
|  |  | Merit ultra-light (5 mg tar, 0.5 mg nicotine) | 30 | ♂/♀ | 32.3 | 11.0 | 19.6 | 4.7 | 60.3 | 20.0 | 2.0 | 0.8 | *30.2* |  | 15.2 | 7.2 | 10.2 | 3.1 | 614.2 | 301.1 | *8.1* | 6.0 | 2.0 | Desktop CReSS |  |
| Dickens *et al* [30] | 2009 | Research cigarette (Virginia tobacco, 8.8 mg nicotine) | 1 | ♂/♀ |  |  |  |  | 95.0 | 2.0 | 3.3 | 0.2 | 29.5 | 2.4 |  |  |  |  | 572.0 |  |  |  |  | Smoker Analyser (SA7) |  |
|  |  |  | 1 | ♂/♀ |  |  |  |  | 72.0 | 1.0 | 1.7 | 0.3 | 40.7 | 1.7 |  |  |  |  | 573.0 |  |  |  |  | Smoker Analyser (SA7) |  |
|  |  |  | 1 | ♂/♀ |  |  |  |  | 63.0 | 13.0 | 1.9 | 0.3 | 35.0 | 2.9 |  |  |  |  | 376.0 |  |  |  |  | Smoker Analyser (SA7) |  |
|  |  |  | 1 | ♂/♀ |  |  |  |  | 57.0 | 1.0 | 1.7 | 0.1 | 34.5 | 1.1 |  |  |  |  | 400.0 |  |  |  |  | Smoker Analyser (SA7) |  |
|  |  |  | 1 | ♂/♀ |  |  |  |  | 119.0 | 7.0 | 2.6 | 0.5 | 27.5 | 3.0 |  |  |  |  | 717.0 |  |  |  |  | Smoker Analyser (SA7) |  |
|  |  |  | 1 | ♂/♀ |  |  |  |  | 73.0 | 4.0 | 1.5 | 0.2 | 35.0 | 0.9 |  |  |  |  | 440.0 |  |  |  |  | Smoker Analyser (SA7) |  |
|  |  |  | 1 | ♂/♀ |  |  |  |  | 55.0 | 5.0 | 3.3 | 0.2 | 29.5 | 2.4 |  |  |  |  | 441.0 |  |  |  |  | Smoker Analyser (SA7) |  |
| Grainge *et al* [28] | 2009 | regular | 130 | ♂/♀ | 31.4 | 10.5 | 13.6 | 6.1 | 56.2 | 13.8 | 1.6 | 0.5 | *35.6* |  | 26.6 | 10.5 | 13.2 | 4.1 | 711.7 | 208.3 | *9.7* |  |  | Portable CReSS |  |
| Moolchan *et al* [31] | 2009 | own brand | 85 | ♂/♀ | 15.3 | 1.2 | 18.6 | 8.5 | 38.5 | 15.6 |  |  |  |  |  |  | 5 |  | 599.5 | 201.6 | *11.1* | 7.0 | 1.2 | CReSS | Fixed puff count |
| Collins *et al* [32] | 2010 | own brand | 89 | ♂/♀ | 15.3 | 1.3 | 18.6 | 9.2 | 41.5 | 1.2 | 1.3 | 0.0 | 53.9 | 2.0 | 14.5 | 0.7 | 15.2 |  | *630.8* |  | *11.7* | 7.3 | 1.3 | CReSS |  |
| O'Connor *et al* [33] | 2010 | own brand (Newport, Marlboro, Camel) | 83 | ♂/♀ | 39.6 | 9.9 | 17.0 | 8.8 | 57.7 | CI 51.8-63.2 | 1.8 | CI 1.57-1.97 | 36.5 | CI 33.4-39.7 | 17.6 | CI 14.8-20.5 | 14.4 | CI 13.1-15.7 | 828.0 | CI 750-905 | *14.1* |  |  | Portable CReSS | Same data as June et al [34] |
|  |  | own brand (Newport, Marlboro, Camel) | 77 | ♂/♀ | 49.0 | 11.9 | 17.9 | 9.3 | 56.7 | Ci 51.2-62.1 | 1.9 | CI 1.70-2.08 | 31.5 | CI 28.5-34.6 | 21.3 | CI 18.6-24.0 | 13.7 | CI 12.5-15.0 | 611.0 | CI 539-682 | *10.9* |  |  | Portable CReSS | Same data as June *et al* [34]*,* Reduced Ignition Propensity type |
| June *et al* [35] | 2011 | Own brand (Number 7, Peter Jackson, Players, DuMaurier) | 42 | ♂/♀ | 32.6 | 12.3 | 18.0 | 7.1 | 59.6 | CI 54.2-64.9 | 1.7 | CI 1.6-1.9 | 36.8 | CI 33.8-39.8 | 19.2 | CI 17.0-21.4 | 14.1 | CI 12.7-15.5 | 834.9 | CI 733.1-936.7 | *15.0* |  |  | Portable CReSS |  |
|  |  | Own brand (Number 7, Peter Jackson, Players, DuMaurier) | 42 | ♂/♀ | 32.6 | 12.3 | 18.0 | 7.1 | 60.3 | CI 54.9-65.8 | 1.7 | CI 1.6-1.9 | 37.2 | CI 34.9-39.6 | 17.9 | CI 15.1-20.6 | 14.2 | CI 12.6-15.9 | 831.7 | CI 745.8-917.6 | *15.0* |  |  | Portable CReSS | Reduced Ignition Propensity type |
| McKee *et al* [36] | 2011 | own brand | 37 | ♂/♀ | 37.7 | 10.6 | 18.4 | 7.96 |  |  |  |  |  |  | 32.9 |  | 9.7 |  |  |  |  | 6.1 | 2.3 | Portable CReSS |  |
| Strasser *et al* [37] | 2011 | own brand | 109 | ♂/♀ | 45.5 | 10.8 | 20.5 | 8.4 |  |  |  |  |  |  |  |  |  |  | 785.1 | 284.9 | *16.1* | 4.9 | 2.1 | Portable CReSS |  |
| Veilleux *et al* [38] | 2011 | own brand | 78 | ♂/♀ | 15.7 | 0.6 | *3.29* |  | 35.2 | 16.2 | 1.1 | 0.4 | 43.6 | 15.6 | 19.2 | 8.9 | 16.5 | 6.1 | 582.7 |  | *1.9* | 2.7 | 1.4 | Portable CReSS | 23.06 cig/week |
| Williams *et al* [39] | 2011 | own brand | 86 | ♂/♀ | 38.0 | 12.0 | 20.0 | 7.7 | 46.2 | SE 0.15 | 1.3 | SE 0.01 | 37.1 | SE 0.1 | 21.0 | SE 0.14 | 12.3 | SE0.15 | 540.5 | SE7.01 | *10.8* | 5.5 | 1.9 | Portable CReSS | control group, same data as Williams *et al* [40] |
| Brinkman *et al* [41] | 2012 | Kent 100 (non-menthol) | 9 | ♂/♀ | 23.0 | 3.5 | 16.9 | SEM 0.7 | 53.9 | SEM 1.0 | 2.2 | SEM 0.1 | 28.1 | SEM 0.6 | 30.6 | SEM 0.6 | 10.8 | SEM 0.5 | 563.0 | SEM20 | *9.5* |  |  | Sodim SPA-D Smoking Puff Analyzer |  |
| Faulkner *et al* [42] | 2012 | own brand | 18 | ♂/♀ | 24.6 | 5.9 | 15.2 | 4.6 | 54.7 | 16.7 | 1.8 | 0.6 | *30.8* |  | 11.9 | 5.1 | 16.5 | 6.3 | *903.2* |  | *13.7* | 4.5 | 2.3 | Portable CReSS |  |
|  |  | Benson & Hedges Light 100 (menthol) | 9 | ♂/♀ | 23.0 | 3.5 | 15.2 | SEM 0.8 | 54.7 | SEM 0.8 | 2.4 | SEM 0.1 | 25.6 | SEM 0.5 | 25.6 | SEM 0.5 | 11.6 | SEM 0.5 | 636.0 | SEM 27 | *9.7* |  |  | Sodim SPA-D Smoking Puff Analyzer |  |
| Perkins *et al* [43] | 2012 | own brand | 94 | ♂/♀ | 26.8 | SE 1 | 19.5 | SE 0.6 |  |  |  |  |  |  |  |  | 12.6 |  | 568.7 |  | *11.1* | 4.7 | SE 0.2 | Portable CReSS |  |
| Matsumoto *et al* [44] | 2013 | Top 10 brands Japan | 101 | ♂/♀ | 40.0 | 11.0 | 18.4 | 7.5 | 54.3 | 14.1 |  |  |  |  |  |  | 14.5 | 3.6 | 767.2 | 259.5 | 14.5 |  |  | Portable CReSS |  |
|  |  | Pianissimo, Mild Seven One (1 mg tar, 0.1 mg nicotine) | 14 | ♂/♀ | 39.2 | 10.2 | 18.7 | 8.3 | 64.6 | 12.3 |  |  |  |  |  |  | 17.9 | 3.2 | 1160.1 | 302.7 | 22.6 |  |  | Portable CReSS |  |
|  |  | Mild Seven One Extra Lights, Caster Mild, Mild Seven Super Lights (3-6 mg tar, 0.3-0.5 mg nicotine) | 38 | ♂/♀ | 40.1 | 12.6 | 17.8 | 7.2 | 56.0 | 12.2 |  |  |  |  |  |  | 15.0 | 3.1 | 810.0 | 179.6 | 14.4 |  |  | Portable CReSS |  |
|  |  | Cabin Mild, Mild Seven Lights, Mild Seven Original (8-10 mg tar, 0.6-0.8 mg nicotine) | 27 | ♂/♀ | 41.9 | 11.2 | 18.2 | 6.6 | 46.9 | 12.3 |  |  |  |  |  |  | 13.4 | 3.4 | 609.4 | 158.7 | 11.4 |  |  | Portable CReSS |  |
|  |  | Hope, Seven Stars (14 mg tar, 1.1-1.2 mg nicotine) | 22 | ♂/♀ | 38.0 | 8.4 | 19.3 | 8.8 | 53.9 | 15.8 |  |  |  |  |  |  | 12.6 | 3.1 | 638.7 | 136.9 | 13.0 |  |  | Portable CReSS |  |
| McClure *et al* [45] | 2013 | own brand | 135 | ♀ | 31.9 | 7.6 | 16.2 | 7 | 49.9 | 15.8 | 1.6 | 0.5 | 32.7 | 9.1 |  |  | 11.7 | 3.9 | *583.8* |  | *9.5* | 4.9 | 2.2 | Portable CReSS |  |
| Strasser *et al* [46] | 2013 | own brand | 78 | ♂/♀ | 38.4 | 11.7 | 18.0 | 8 | *54.7* |  | 1.7 | 0.1 | *32.2* |  | 30.1 | 2.1 | 11.2 | 0.6 | 612.6 | 74.6 | *11.0* | 5.8 | 1.8 | Portable CReSS | Day 1 - cigarette 1 |
|  |  | own brand | 78 | ♂/♀ | 38.4 | 11.7 | 18.0 | 8 | *46.7* |  | 1.7 | 0.1 | *27.4* |  | 33.7 | 3.2 | 11.1 | 0.8 | 517.9 | 36.8 | *9.3* | 5.8 | 1.8 | Portable CReSS | Day 1 - cigarette 2 |
|  |  | own brand | 78 | ♂/♀ | 38.4 | 11.7 | 18.0 | 8 | *44.5* |  | 1.6 | 0.1 | *27.8* |  | 31.4 | 3.0 | 12.6 | 0.7 | 560.7 | 42.7 | *10.1* | 5.8 | 1.8 | Portable CReSS | Day 5 |
| Tidey *et al* [47] | 2013 | own brand | 26 | ♂/♀ | 44.5 | 10.9 | 23.5 | 6.5 |  |  |  |  |  |  |  |  |  |  | 1451.0 | 835.0 | *34.1* | 6.7 | 1.8 | Desktop CReSS |  |
| Hammond *et al* [48] | 2014 | own brand | 72 | ♂/♀ | 37.2 | 11.9 | 16.1 | 6.3 | 63.5 | 22.4 |  |  |  |  |  |  | 16.1 | 6.3 | 977.9 | 420.8 | *15.7* | 4.9 | 2.3 | Portable CReSS |  |
|  |  | Quest 1 (0.6 mg nicotine) | 71 | ♂/♀ | 37.2 | 11.9 | 14.5 | 4.6 | 64.3 | 22.3 |  |  |  |  |  |  | 14.5 | 4.6 | 910.1 | 520.3 | *13.2* | 4.8 | 2.2 | Portable CReSS |  |
|  |  | Quest 2 (0.3 mg nicotine) | 71 | ♂/♀ | 37.2 | 11.9 | 13.8 | 4.3 | 64.2 | 17.2 |  |  |  |  |  |  | 13.8 | 4.3 | 834.2 | 190.4 | *11.5* | 4.8 | 2.1 | Portable CReSS |  |
|  |  | Quest 3 (0.05 mg nicotine) | 72 | ♂/♀ | 37.2 | 11.9 | 13.2 | 4.5 | 69.3 | 18.8 |  |  |  |  |  |  | 13.2 | 4.5 | 890.7 | 353.6 | *11.8* | 4.4 | 2.3 | Portable CReSS |  |
| Norton *et al* [49] | 2014 | own brand | 16 | ♂/♀ | 45.5 | SE 3.5 | 16.1 | SE 1.4 | 67.5 | SE 6.3 | 3.0 | SE 1 | 36.1 | SE 1.8 | 21.3 | SE 6.2 | 13.2 | SE 1.1 | 829.0 | SE 51.4 | *13.3* |  |  | Portable CReSS |  |
| Schneider *et al* [50] | 2014 | own brand | 25 | ♂/♀ | 45.9 | 12.4 | 16.7 | 4.6 | 62.2 | 18.5 | 1.7 | 0.5 | *36.8* |  | 18.7 | 7.7 | 10.2 | 2.7 | *635.5* |  | *10.6* | 5.6 | 2.0 | Portable CReSS |  |
|  |  | own brand | 25 | ♂/♀ | 45.9 | 12.4 | 16.7 | 4.6 | 58.8 | 18.3 | 1.6 | 0.4 | *36.3* |  | 19.2 | 7.6 | 11.4 | 2.7 | *667.5* |  | *11.2* | 5.6 | 2.0 | Portable CReSS |  |
|  |  | own brand | 23 | ♂/♀ | 39.1 | 13.8 | 15.6 | 4.22 | 55.3 | 18.5 | 1.7 | 0.4 | *31.8* |  | 14.3 | 5.0 | 14.2 | 4.7 | *783.6* |  | *12.2* | 4.2 | 1.9 | Portable CReSS |  |
|  |  | own brand | 23 | ♂/♀ | 39.1 | 13.8 | 15.6 | 4.22 | 54.8 | 16.9 | 1.7 | 0.5 | *32.2* |  | 15.0 | 5.5 | 13.9 | 5.6 | *763.9* |  | *11.9* | 4.2 | 1.9 | Portable CReSS | after 10 minutes exercise |
| Appleton *et al* [51] | 2015 | own brand (>13 mg tar) | 20 | ♂ | 35.3 | 6.1 | 10-30 |  | 69.7 | 18.6 | 2.3 | 0.6 | *30.3* |  | 38.6 | 10.8 | 9.8 | 2.3 | *683.1* |  | *6.8* |  |  | Portable CReSS | Total volume if 10 cig/day |
|  |  | Test cigarette High Tar (~15 mg tar) | 20 | ♂ | 35.4 | 6.4 | 22.4-24.0 |  | 59.4 | 18.3 | 2.0 | 0.6 | *29.4* |  | 37.2 | 10.2 | 8.9 | 2.2 | *528.4* |  | *11.8* |  |  | Portable CReSS | Total volume if 22.4 cig/day |
|  |  | Test Cigarette Low tar (~7 mg tar) | 19 | ♂ | 35.1 | 6.2 | 20.5-22.6 |  | 69.3 | 19.2 | 2.4 | 0.7 | *29.5* |  | 33.3 | 9.0 | 9.5 | 2.5 | *658.4* |  | *13.5* |  |  | Portable CReSS | Total volume if 20.5 cig/day |
| Brinkman *et al* [52] | 2015 | GPC Full flavor and GPC Ultra Light | 24 | ♂/♀ | 18-30 |  | >20 |  | 55.6 | CI 53.71-57.49 | 2.1 | CI 2.042-2.198 | 27.2 | CI 26.22-28.21 | 19.3 | CI 18.48-20.14 | *11.6* |  | 643.8 | CI 627.2-660.9 | *12.9* |  |  | Sodim SPA-D Smoking Puff Analyzer | Based on puff profiles of Brinkman *et al* [41] |
|  |  | GPC Full flavor (15.2 mg tar, 0.97 mg nicotine) | 24 | ♂/♀ | 18-30 |  | >20 |  | 51.9 | CI 49.42-54.45 | 2.0 | CI 1.9-2.13 | 26.7 | CI 25.40-28.04 | 21.0 | CI 19.87-22.20 | *11.5* |  | 598.5 | CI 579.84-617.77 | *12.0* |  |  | Sodim SPA-D Smoking Puff Analyzer | Based on puff profiles of Brinkman *et al* [41] |
|  |  | GPC Ultra Light (5.8 mg tar, 0.51 mg nicotine) | 24 | ♂/♀ | 18-30 |  | >20 |  | 59.6 | CI 56.94-62.37 | 2.2 | CI 2.14-2.34 | 27.7 | CI 26.27-29.27 | 17.7 | CI 16.59-18.86 | *11.6* |  | 693.4 | CI 666.79-721.06 | *13.9* |  |  | Sodim SPA-D Smoking Puff Analyzer | Based on puff profiles of Brinkman *et al* [41] |
| Chung *et al* [53] | 2015 | own brand - white | 26 | ♂ | 39.2 | 10.1 | >12 |  | 57.2 | SE 2.69 | 1.6 | SE 0.06 | 37.1 | SE 1.70 | 25.0 | SE 1.52 | 12.3 | SE 0.61 | *700.8* |  | *8.4* | 5.8 | 2.1 | Portable CReSS |  |
|  |  | own brand- korean | 27 | ♂ | 37.3 | 9.7 | 12.0 | 1.82 | 59.6 | SE 3 | 1.5 | SE 0.07 | 42.1 | SE 1.94 | 13.5 | SE 1.7 | 13.8 | SE 0.69 | *820.6* |  | *9.8* | 4.6 | 2.1 | Portable CReSS |  |
| Gass *et al* [54] | 2016 | own brand | 107 | ♂/♀ | 25.0 | 5.9 | 2.7 | 2.2 | 31.4 | 13.0 | 1.2 | 0.5 | 26.8 | 7.9 | 28.8 | 45.4 | 13.5 | 4.5 | 413.6 | 182.2 | *1.1* | 1.1 | 1.5 | Portable CReSS |  |
| Krebs *et al* [55] | 2016 | own brand | 332 | ♂/♀ | 37.6 | 11.6 | 16.5 | 6.2 | 48.2 | 14.9 | 1.6 | 0.4 | 34.0 | 1.3 | 26.6 | 23.8 | 12.2 | 4.8 | *588.0* |  | 5526.6 | 4.4 | 2.3 | SPA-M | Pennsylvania Adult Smoking Study, same study as Chen *et al* [56] |
| Mercincagave *et al* [57] | 2016 | Own brand | 63 | ♂/♀ | 40.7 | 12.7 | 19.3 | CI 17.68-20.98 | 56.5 | CI 52.45-60.47 | 1.8 | CI 1.63-1.89 | *32.1* |  | 24.4 | 21.34-27.40 | 13.8 | CI 12.63-14.88 | 756.0 | CI 690.68-821.34 | *14.6* | 5.6 | 1.9 | Portable CReSS | same study as Mercincagave *et al* [58] |
|  |  | Quest 1 (0.6 mg nicotine) | 63 | ♂/♀ | 40.7 | 12.7 | 22.6 | CI 20.93-24.36 | 52.2 | CI 48.13-56.30 | 1.7 | CI 1.53-1.79 | *31.5* |  | 20.9 | CI 18.36-23.46 | 10.8 | CI 9.87-11.81 | 541.0 | CI 489.42-592.51 | *12.2* | 5.6 | 1.9 | Portable CReSS | same study as Mercincagave *et al* [58] |
|  |  | Quest 2 (0.3 mg nicotine) | 63 | ♂/♀ | 40.7 | 12.7 | 24.2 | CI 22.35-26.01 | 52.7 | CI 48.91-56.37 | 1.8 | CI 1.61-1.88 | *30.1* |  | 19.1 | CI 16.51-21.58 | 10.9 | CI 9.92-11.91 | 545.1 | CI 499.34-590.86 | *13.2* | 5.6 | 1.9 | Portable CReSS | same study as Mercincagave *et al* [58] |
|  |  | Quest 3 (0.05 mg nicotine) | 63 | ♂/♀ | 40.7 | 12.7 | 20.6 | CI 18.53-22.58 | 61.1 | CI 57.18-65.08 | 1.9 | CI 1.79-2.05 | *31.8* |  | 17.5 | CI 15.04-19.91 | 11.8 | CI 10.63-12.88 | 691.1 | CI 632.34-749.91 | *14.2* | 5.6 | 1.9 | Portable CReSS | same study as Mercincagave *et al* [58] |
| Pulcu *et al* [59] | 2016 | own brand | 110 | ♂/♀ | 28.2 | 9.4 | 15.8 | 6.2 |  |  |  |  |  |  | 26.6 | 11.4 | 14.4 | 4.0 |  |  |  |  |  | Portable CReSS |  |
| Ross *et al* [60] | 2016 | own brand | 59 | ♂/♀ | 34.9 | 10.1 | 14.0 | 6.4 | 45.9 | 16.6 | 1.5 | 0.1 | 31.3 | 8.4 | 21.4 | 9.4 | 12.4 | 3.9 | 536.4 | 117.0 | *7.5* | 5.1 | 2.0 | Portable CReSS | first cigarette of the day |
|  |  |  | 59 | ♂/♀ | 34.9 | 10.1 | 14.0 | 6.4 | 46.6 | 16.0 | 1.5 | 0.5 | 32.1 | 8.8 | 19.4 | 8.4 | 12.9 | 4.0 | 554.5 | 164.7 | *7.8* | 5.1 | 2.0 | Portable CReSS | after 4 hours ad libitum smoking |
|  |  |  | 30 | ♂ | 35.5 | 11.1 | 14.6 | 6.9 | 50.9 | 17.7 | 1.7 | 0.5 | 31.4 | 10.5 | 21.2 | 7.3 | 11.4 | 2.8 | 559.5 | 200.9 | *8.2* | 4.5 | 2.0 | Portable CReSS | first cigarette of the day |
|  |  |  | 30 | ♂ | 35.5 | 11.1 | 14.6 | 6.9 | 52.2 | 17.6 | 1.7 | 0.5 | 31.9 | 11.0 | 18.7 | 8.4 | 12.2 | 3.2 | 582.2 | 171.8 | *8.5* | 4.5 | 2.0 | Portable CReSS | after 4 hours ad libitum smoking |
|  |  |  | 29 | ♀ | 34.4 | 9.2 | 13.4 | 5.9 | 40.7 | 13.7 | 1.4 | 0.4 | 31.1 | 5.8 | 21.6 | 11.3 | 13.4 | 4.6 | 512.5 | 148.1 | *6.9* | 5.7 | 1.8 | Portable CReSS | first cigarette of the day |
|  |  |  | 29 | ♀ | 34.4 | 9.2 | 13.4 | 5.9 | 40.9 | 12.0 | 1.3 | 0.4 | 32.2 | 5.9 | 20.0 | 8.4 | 13.7 | 4.7 | 526.7 | 155.3 | *7.1* | 5.7 | 1.8 | Portable CReSS | after 4 hours ad libitum smoking |
| Ross *et al* [61] | 2016 | own brand (non-menthol) | 58 | ♂/♀ | 40.0 | 13.2 | 11.8 | 5.62 | *82.9* |  | 2.1 | 0.5 | 39.9 | 8.3 |  |  | 14.6 | 4.6 | *1213.3* |  | *14.4* | 4.0 | 2.3 | Portable CReSS |  |
| Tidey *et al* [62] | 2016 | own brand | 23 | ♂/♀ | 45.0 | 11.1 | 23.3 | 6.2 | 44.0 |  | 1.4 |  | *31.4* |  | 35.0 |  | 11.0 |  | 500.0 |  | *11.7* |  |  | Portable CReSS | Graph data, originates from Tidey *et al* [47] |
| Farris *et al* [63] | 2017 | own brand | 64 | ♂/♀ | 44.3 | 9.5 | 14.8 | 5.47 | 63.2 | 24.4 | 1.8 | 0.6 | *35.9* |  | 14.3 | 5.4 | 20.4 | 7.4 | *1290.1* |  | *19.1* | 4.6 | 1.5 | Portable CReSS |  |
| Higgins *et al* [64] | 2017 | SPECTRUM 15.8 mg nicotine/g tobacco | 26 | ♂/♀ | 36.7 | 10.8 | 17.5 | 10.15 | 38.9 | 15.1 | 1.4 | 0.4 | *28.8* |  | 19.9 | 9.3 | 15.0 | 8.3 | 463.8 | 211.9 | *8.1* | 5.2 | 2.7 | Desktop CReSS |  |
|  |  | SPECTRUM 5.2 mg nicotine/g tobacco | 26 | ♂/♀ | 36.7 | 10.8 | 17.5 | 10.16 | 35.6 | 16.1 | 1.2 | 0.5 | *28.9* |  | 27.8 | 24.2 | 12.0 | 5.0 | 370.7 | 212.2 | *6.5* | 5.2 | 2.7 | Desktop CReSS |  |
|  |  | SPECTRUM 2.4 mg nicotine/g tobacco | 26 | ♂/♀ | 36.7 | 10.8 | 17.5 | 10.17 | 41.5 | 12.6 | 1.4 | 0.4 | *29.9* |  | 21.0 | 8.2 | 11.3 | 3.2 | 444.4 | 200.1 | *7.8* | 5.2 | 2.7 | Desktop CReSS |  |
|  |  | SPECTRUM 0.4 mg nicotine/g tobacco | 26 | ♂/♀ | 36.7 | 10.8 | 17.5 | 10.18 | 38.6 | 12.5 | 1.4 | 0.4 | *28.0* |  | 21.3 | 6.1 | 11.7 | 3.1 | 411.9 | 184.2 | *7.2* | 5.2 | 2.7 | Desktop CReSS |  |
| Hsu *et al* [65] | 2017 | own brand (non-menthol) | 34 | ♂/♀ | 41.4 | 10.9 | >10 |  | 52.7 | 18.0 | 1.6 | 0.8 | *32.9* |  | 21.0 | 14.7 | 12.3 | 6.6 | 690.3 | 547.8 | *6.9* | 5.4 | 2.7 | CReSS |  |
|  |  | own brand (menthol) | 71 | ♂/♀ | 43.8 | 9.3 | >10 |  | 55.7 | 18.5 | 1.8 | 0.6 | *30.9* |  | 20.0 | 12.7 | 12.6 | 7.5 | 691.7 | 455.9 | *6.9* | 4.8 | 2.1 | CReSS |  |
| Watson *et al* [66] | 2017 | Kent 100 (non-menthol) | 42 | ♂/♀ | 35.0 |  | 23.0 |  | 47.7 | SEM 1.5 | 1.2 | SEM 0.03 | *40.8* |  |  |  | 14.4 | SEM 0.5 | *686.9* |  | *15.8* |  |  | Portable CReSS |  |
|  |  | Benson & Hedges Light 100 (menthol) | 42 | ♂/♀ | 35.0 |  | 22.0 |  | 52.2 | SEM 2.7 | 1.2 | SEM 0.03 | *42.4* |  |  |  | 15.1 | SEM 0.45 | *788.2* |  | *17.3* |  |  | Portable CReSS |  |
| Bergeria *et al* [67] | 2018 | own brand | 89 | ♀ | 29.7 | 6.4 | 15.3 | 6.1 | 48.3 | 2.9 | 1.3 | 0.1 | 36.5 | 1.4 | 20.5 | 1.0 | 14.8 | 0.6 | *716.4* |  | *11.0* | 4.6 | 2.2 | Desktop CReSS |  |
|  |  | own brand | 20 | ♀ | 30.3 | 4.9 | 12.6 | 5.4 | 48.2 | 5.0 | 1.3 | 0.1 | 35.1 | 2.4 | 23.7 | 2.6 | 14.0 | 1.4 | *674.9* |  | *8.5* | 4.4 | 2.2 | Desktop CReSS | pregnant |
| Chen *et al* [56] | 2018 | own brand | 326 | ♂/♀ | 37.6 | 11.6 | 16.5 | 8.1 | 48.3 | 14.9 | 1.6 | 0.4 | *30.2* |  |  |  | 7.6 | 4.6 | 360.0 | 222.0 | 5.5 | 4.4 | 2.3 | SPA-M | Pennsylvania Adult Smoking Study, same data as Krebs *et al* [55] |
| Kim *et al* [68] | 2018 | own brand | 300 | ♂/♀ | ~30 |  | 0->20 |  | median 60.6 | IQR 49.9-73.9 | median 1.5528 | IQR 1.243-1.923 | median 40.4 | IQR 32.1-51.1 | median 8.8289 | IQR 6.5286-1.11961 | median 15.5 | IQR 13.5-19.0 | *969.6* |  |  |  |  | Portable CReSS | IQR interquartile range |
|  |  |  | 250 | ♂ | ~30 |  | 0->20 |  | median 32.7 | IQR 52.7-75.5 | median 1.6 | 1.2-1.9 | median 41.2 | IQR 33.1-52.2 | median 8.9 | IQR 6.5-11.2 | median 15.5 | IQR 13-19 | *971.9* |  |  |  |  | Portable CReSS |  |
|  |  |  | 50 | ♀ | ~30 |  | 0->20 |  | median 53.5 | IQR 42-64.2 | median 1.4 | 1.2-1.8 | median 36.4 | IQR 30-43.7 | median 8.3 | IQR 6.2-11 | median 17.5 | IQR 15-21 | *936.3* |  |  |  |  | Portable CReSS |  |
| Mercincagave *et al* [58] | 2018 | own brand (non menthol) | 84 | ♂/♀ | 43.0 | 12.1 | 13.9 | CI 12.5-15.2 | 52.7 | CI 48.1-57.4 | 1.7 | CI 1.6-1.8 | *31.0* |  | 26.1 | CI 23.2-28.9 | 14.7 | CI 13.6-15.9 | 744.0 | CI 681-806 | *10.3* | 5.5 | 1.8 | CReSS |  |
|  |  | SPECTRUM 400 (9 mg tar, 0.26 mg nicotine) | 84 | ♂/♀ | 43.0 | 12.1 | 16.5 | CI 14.9-18.1 | 49.4 | CI 44.4-54.3 | 1.7 | CI 1.5-1.8 | *29.1* |  | 22.3 | CI 19.7-24.8 | 11.2 | CI 10.4-11.9 | 537.0 | CI 479-595 | *8.9* | 5.5 | 1.8 | CReSS | Research cigarette with reduced nicotine content (5.2mg nicotine/g tobacco) |
|  |  | SPECTRUM 200 (9 mg tar, 0.07 mg nicotine) | 84 | ♂/♀ | 43.0 | 12.1 | 14.8 | CI 13.2-16.5 | 50.1 | CI 45.5-54.6 | 1.8 | CI 1.7-2.0 | *27.8* |  | 20.7 | CI 18.2-23.2 | 12.3 | CI 11.5-13.1 | 598.0 | CI 547-649 | *8.9* | 5.5 | 1.8 | CReSS | Research cigarette with reduced nicotine content (1mg nicotine/g tobacco) |
| Shiffman *et al* [69] | 2018 | own brand | 190 | ♂/♀ | 38.0 | 13.8 | 2.0 |  | *39.6* |  | *1.5* |  | *25.7* |  |  |  | 15.8 | 6.0 | 625.6 | 209.8 | *1.2* |  |  | Portable CReSS | bought, total puff time known |
|  |  | own brand | 190 | ♂/♀ | 38.0 | 13.8 | 3.3 |  | *38.4* |  | *1.5* |  | *25.7* |  |  |  | 15.1 | 6.1 | 581.2 | 227.6 | *1.9* |  |  | Portable CReSS | provided for free, total puff time known |
| Vansickel *et al* [70] | 2018 | own brand | 13 | ♂/♀ | 45.0 | 11.0 | 15.4 | 4 | 44.1 | 10.5 | 1.7 | 0.4 | 28.6 | 6.1 |  |  |  |  |  |  |  |  |  | Sodim SPA-D Smoking Puff Analyzer |  |
| Yuki *et al* [71] | 2018 | own brand (~6.5 mg tar) | 20 | ♂/♀ | 30.9 | 12.5 | 17.3 | 6.6 | 58.5 | 20.2 | 1.6 | 0.4 | *36.1* |  |  |  | 14.6 | 4.2 | 858.0 | 369.0 | *14.8* | 4.3 | 1.7 | Portable CReSS | Baseline |
|  |  | own brand (~6.5 mg tar) | 20 | ♂/♀ | 30.9 | 12.5 | 17.3 | 6.6 | 58.8 | 19.0 | 1.8 | 0.7 | *32.3* |  |  |  | 14.6 | 3.8 | 857.0 | 296.0 | *14.8* | 4.3 | 1.7 | Portable CReSS | day 1 |
|  |  | own brand (~6.5 mg tar) | 20 | ♂/♀ | 30.9 | 12.5 | 17.3 | 6.6 | 62.8 | 19.2 | 1.8 | 0.5 | *35.3* |  |  |  | 15.2 | 3.4 | 989.0 | 425.0 | *17.1* | 4.3 | 1.7 | Portable CReSS | day 3 |
|  |  | own brand (~6.5 mg tar) | 20 | ♂/♀ | 30.9 | 12.5 | 17.3 | 6.6 | 56.0 | 14.4 | 1.6 | 0.4 | *34.6* |  |  |  | 14.2 | 3.4 | 847.0 | 292.0 | *14.7* | 4.3 | 1.7 | Portable CReSS | day 5 |
| Watson *et al* [72] | 2019 | own brand | 163 | ♂/♀ | 42.4 | 18-66 | 16.1 | 6-40 | 54.1 | CI 51.5-56.7 | 1.5 | CI 1.48-1.59 | *35.4* |  | 16.8 | CI 15.2-18.5 | 11.0 | CI 9.8-12.1 | *595.1* |  | *9.6* |  |  | CReSS | day 1 |
|  |  | own brand | 163 | ♂/♀ | 42.4 | 18-66 | 16.1 | 6-40 | 53.6 | CI 51.1-56.1 | 1.5 | CI 1.47-1.58 | *35.3* |  | 16.3 | CI 14.7-17.9 | 14.9 | CI 13.4-16.5 | *798.6* |  | *12.9* |  |  | CReSS | day 2 |

Calculated values in *italic*. SD = standard deviation, CI = 95% Confidence interval, SEM = Standard error of the mean, SE = Standard error, ♀ female, ♂ male, CRSSPT = Clinical Research Support System, Plowshare Technologies, CReSS = CReSSmicro™ (Clinical Research Support System), Plowshare Technologies. Blue: highest value. Yellow: lowest value. ^$^ Data originates from ISO/TR 17219:2013 (stated as personal communication) [73]

References

1. Buchhalter, A.R.; Eissenberg, T. Preliminary evaluation of a novel smoking system: effects on subjective and physiological measures and on smoking behavior. Nicotine Tob Res 2000, 2, 39-43, doi:10.1080/14622200050011286.

2. Djordjevic, M.V.; Stellman, S.D.; Zang, E. Doses of nicotine and lung carcinogens delivered to cigarette smokers. J. Natl. Cancer Inst. 2000, 92, 106-111, doi:10.1093/jnci/92.2.106.

3. Breland, A.B.; Buchhalter, A.R.; Evans, S.E.; Eissenberg, T. Evaluating acute effects of potential reduced-exposure products for smokers: clinical laboratory methodology. Nicotine Tob Res 2002, 4 Suppl 2, S131-140, doi:10.1080/1462220021000032780.

4. Breland, A.B.; Evans, S.E.; Buchhalter, A.R.; Eissenberg, T. Acute effects of Advance: a potential reduced exposure product for smokers. Tob. Control 2002, 11, 376-378, doi:10.1136/tc.11.4.376.

5. Dixon, M.; Kochhar, N.; Prasad, K.; Shepperd, J.; Warburton, D.M. The influence of changing nicotine to tar ratios on human puffing behaviour and perceived sensory response. Psychopharmacology 2003, 170, 434-442, doi:10.1007/s00213-003-1541-8.

6. Lee, E.M.; Malson, J.L.; Waters, A.J.; Moolchan, E.T.; Pickworth, W.B. Smoking topography: reliability and validity in dependent smokers. Nicotine Tob Res 2003, 5, 673-679, doi:10.1080/1462220031000158645.

7. Aung, A.T.; Pickworth, W.B.; Moolchan, E.T. History of marijuana use and tobacco smoking topography in tobacco-dependent adolescents. Addict. Behav. 2004, 29, 699-706, doi:10.1016/j.addbeh.2004.02.012.

8. Hughes, J.R.; Hecht, S.S.; Carmella, S.G.; Murphy, S.E.; Callas, P. Smoking behaviour and toxin exposure during six weeks use of a potential reduced exposure product: Omni. Tob. Control 2004, 13, 175-179, doi:10.1136/tc.2003.005439.

9. Lee, E.M.; Malson, J.L.; Moolchan, E.T.; Pickworth, W.B. Quantitative comparisons between a nicotine delivery device (Eclipse) and conventional cigarette smoking. Nicotine Tob Res 2004, 6, 95-102, doi:10.1080/14622200310001656911.

10. Strasser, A.A.; Pickworth, W.B.; Patterson, F.; Lerman, C. Smoking topography predicts abstinence following treatment with nicotine replacement therapy. Cancer Epidemiol. Biomarkers Prev. 2004, 13, 1800-1804.

11. Wood, T.; Wewers, M.E.; Groner, J.; Ahijevych, K. Smoke constituent exposure and smoking topography of adolescent daily cigarette smokers. Nicotine Tob Res 2004, 6, 853-862, doi:10.1080/1462220042000282537.

12. Zimmerman, D.M.; Sehnert, S.S.; Epstein, D.H.; Pickworth, W.B.; Robinson, M.L.; Moolchan, E.T. Smoking topography and trajectory of asthmatic adolescents requesting cessation treatment. Prev. Med. 2004, 39, 940-942, doi:10.1016/j.ypmed.2004.03.032.

13. Hammond, D.; Fong, G.T.; Cummings, K.M.; Hyland, A. Smoking topography, brand switching, and nicotine delivery: results from an in vivo study. Cancer Epidemiol. Biomarkers Prev. 2005, 14, 1370-1375, doi:10.1158/1055-9965.EPI-04-0498.

14. Hitsman, B.; Spring, B.; Wolf, W.; Pingitore, R.; Crayton, J.W.; Hedeker, D. Effects of acute tryptophan depletion on negative symptoms and smoking topography in nicotine-dependent schizophrenics and nonpsychiatric controls. Neuropsychopharmacology 2005, 30, 640-648, doi:10.1038/sj.npp.1300651.

15. Strasser, A.A.; Ashare, R.L.; Kozlowski, L.T.; Pickworth, W.B. The effect of filter vent blocking and smoking topography on carbon monoxide levels in smokers. Pharmacol. Biochem. Behav. 2005, 82, 320-329, doi:10.1016/j.pbb.2005.09.001.

16. Tidey, J.W.; Rohsenow, D.J.; Kaplan, G.B.; Swift, R.M. Cigarette smoking topography in smokers with schizophrenia and matched non-psychiatric controls. Drug Alcohol Depend. 2005, 80, 259-265, doi:10.1016/j.drugalcdep.2005.04.002.

17. Breland, A.B.; Kleykamp, B.A.; Eissenberg, T. Clinical laboratory evaluation of potential reduced exposure products for smokers. Nicotine Tob Res 2006, 8, 727-738, doi:10.1080/14622200600789585.

18. Franken, F.H.; Pickworth, W.B.; Epstein, D.H.; Moolchan, E.T. Smoking rates and topography predict adolescent smoking cessation following treatment with nicotine replacement therapy. Cancer Epidemiol. Biomarkers Prev. 2006, 15, 154-157, doi:10.1158/1055-9965.EPI-05-0167.

19. Hammond, D.; Fong, G.T.; Cummings, K.M.; O'Connor, R.J.; Giovino, G.A.; McNeill, A. Cigarette yields and human exposure: a comparison of alternative testing regimens. Cancer Epidemiol. Biomarkers Prev. 2006, 15, 1495-1501, doi:10.1158/1055-9965.EPI-06-0047.

20. Kassel, J.D.; Greenstein, J.E.; Evatt, D.P.; Wardle, M.C.; Yates, M.C.; Veilleux, J.C.; Eissenberg, T. Smoking topography in response to denicotinized and high-yield nicotine cigarettes in adolescent smokers. J. Adolesc. Health 2007, 40, 54-60, doi:10.1016/j.jadohealth.2006.08.006.

21. Melikian, A.A.; Djordjevic, M.V.; Hosey, J.; Zhang, J.; Chen, S.; Zang, E.; Muscat, J.; Stellman, S.D. Gender differences relative to smoking behavior and emissions of toxins from mainstream cigarette smoke. Nicotine Tob Res 2007, 9, 377-387, doi:10.1080/14622200701188836.

22. Djordjevic, M.V.; Fan, J.; Ferguson, S.; Hoffmann, D. Self-regulation of smoking intensity. Smoke yields of the low-nicotine, low-'tar' cigarettes. Carcinogenesis 1995, 16, 2015-2021, doi:10.1093/carcin/16.9.2015.

23. Strasser, A.A.; Lerman, C.; Sanborn, P.M.; Pickworth, W.B.; Feldman, E.A. New lower nicotine cigarettes can produce compensatory smoking and increased carbon monoxide exposure. Drug Alcohol Depend. 2007, 86, 294-300, doi:10.1016/j.drugalcdep.2006.06.017.

24. Strasser, A.A.; Malaiyandi, V.; Hoffmann, E.; Tyndale, R.F.; Lerman, C. An association of CYP2A6 genotype and smoking topography. Nicotine Tob Res 2007, 9, 511-518, doi:10.1080/14622200701239605.

25. Attwood, A.S.; O'Sullivan, H.; Leonards, U.; Mackintosh, B.; Munafo, M.R. Attentional bias training and cue reactivity in cigarette smokers. Addiction 2008, 103, 1875-1882, doi:10.1111/j.1360-0443.2008.02335.x.

26. Collins, C.C.; Lippmann, B.M.; Lo, S.J.; Moolchan, E.T. Time spent with smoking parents and smoking topography in adolescents. Addict. Behav. 2008, 33, 1594-1597, doi:10.1016/j.addbeh.2008.07.003.

27. Rees, V.W.; Wayne, G.F.; Connolly, G.N. Puffing style and human exposure minimally altered by switching to a carbon-filtered cigarette. Cancer Epidemiol. Biomarkers Prev. 2008, 17, 2995-3003, doi:10.1158/1055-9965.EPI-07-2533.

28. Grainge, M.J.; Shahab, L.; Hammond, D.; O'Connor, R.J.; McNeill, A. First cigarette on waking and time of day as predictors of puffing behaviour in UK adult smokers. Drug Alcohol Depend. 2009, 101, 191-195, doi:10.1016/j.drugalcdep.2009.01.013.

29. Blank, M.D.; Disharoon, S.; Eissenberg, T. Comparison of methods for measurement of smoking behavior: mouthpiece-based computerized devices versus direct observation. Nicotine Tob Res 2009, 11, 896-903, doi:10.1093/ntr/ntp083.

30. Dickens, C.; McGrath, C.; Warren, N.; Biggs, P.; McAughey, J. Puffing and inhalation behaviour in cigarette smoking: Implications for particle diameter and dose. Journal of Physics: Conference Series 2009, 151, 012019.

31. Moolchan, E.T.; Parzynski, C.S.; Jaszyna-Gasior, M.; Collins, C.C.; Leff, M.K.; Zimmerman, D.L. A link between adolescent nicotine metabolism and smoking topography. Cancer Epidemiol. Biomarkers Prev. 2009, 18, 1578-1583, doi:10.1158/1055-9965.EPI-08-0592.

32. Collins, C.C.; Epstein, D.H.; Parzynski, C.S.; Zimmerman, D.; Moolchan, E.T.; Heishman, S.J. Puffing behavior during the smoking of a single cigarette in tobacco-dependent adolescents. Nicotine Tob Res 2010, 12, 164-167, doi:10.1093/ntr/ntp176.

33. O'Connor, R.J.; Rees, V.W.; Norton, K.J.; Cummings, K.M.; Connolly, G.N.; Alpert, H.R.; Sjodin, A.; Romanoff, L.; Li, Z.; June, K.M., et al. Does switching to reduced ignition propensity cigarettes alter smoking behavior or exposure to tobacco smoke constituents? Nicotine Tob Res 2010, 12, 1011-1018, doi:10.1093/ntr/ntq139.

34. June, K.M.; Norton, K.J.; Rees, V.W.; O'Connor, R.J. Influence of measurement setting and home smoking policy on smoking topography. Addict. Behav. 2012, 37, 42-46, doi:10.1016/j.addbeh.2011.07.039.

35. June, K.M.; Hammond, D.; Sjodin, A.; Li, Z.; Romanoff, L.; O'Connor, R.J. Cigarette ignition propensity, smoking behavior, and toxicant exposure: A natural experiment in Canada. Tob. Induc. Dis. 2011, 9, 13, doi:10.1186/1617-9625-9-13.

36. McKee, S.A.; Sinha, R.; Weinberger, A.H.; Sofuoglu, M.; Harrison, E.L.; Lavery, M.; Wanzer, J. Stress decreases the ability to resist smoking and potentiates smoking intensity and reward. J Psychopharmacol 2011, 25, 490-502, doi:10.1177/0269881110376694.

37. Strasser, A.A.; Benowitz, N.L.; Pinto, A.G.; Tang, K.Z.; Hecht, S.S.; Carmella, S.G.; Tyndale, R.F.; Lerman, C.E. Nicotine metabolite ratio predicts smoking topography and carcinogen biomarker level. Cancer Epidemiol. Biomarkers Prev. 2011, 20, 234-238, doi:10.1158/1055-9965.EPI-10-0674.

38. Veilleux, J.C.; Kassel, J.D.; Heinz, A.J.; Braun, A.; Wardle, M.C.; Greenstein, J.; Evatt, D.P.; Conrad, M. Predictors and sequelae of smoking topography over the course of a single cigarette in adolescent light smokers. J. Adolesc. Health 2011, 48, 176-181, doi:10.1016/j.jadohealth.2010.06.015.

39. Williams, J.M.; Gandhi, K.K.; Lu, S.E.; Kumar, S.; Steinberg, M.L.; Cottler, B.; Benowitz, N.L. Shorter interpuff interval is associated with higher nicotine intake in smokers with schizophrenia. Drug Alcohol Depend. 2011, 118, 313-319, doi:10.1016/j.drugalcdep.2011.04.009.

40. Williams, J.M.; Gandhi, K.K.; Lu, S.E.; Steinberg, M.L.; Benowitz, N.L. Nicotine intake and smoking topography in smokers with bipolar disorder. Bipolar Disord 2012, 14, 618-627, doi:10.1111/j.1399-5618.2012.01047.x.

41. Brinkman, M.C.; Chuang, J.C.; Gordon, S.M.; Kim, H.; Kroeger, R.R.; Polzin, G.M.; Richter, P.A. Exposure to and deposition of fine and ultrafine particles in smokers of menthol and nonmenthol cigarettes. Inhal. Toxicol. 2012, 24, 255-269, doi:10.3109/08958378.2012.667218.

42. Faulkner, G.E.; Arbour-Nicitopoulos, K.P.; Hsin, A. Cutting Down One Puff at a Time: The Acute Effects of Exercise on Smoking Behaviour. Journal of Smoking Cessation 2012, 5, 130-135, doi:10.1375/jsc.5.2.130.

43. Perkins, K.A.; Karelitz, J.L.; Giedgowd, G.E.; Conklin, C.A. The reliability of puff topography and subjective responses during ad lib smoking of a single cigarette. Nicotine Tob Res 2012, 14, 490-494, doi:10.1093/ntr/ntr150.

44. Matsumoto, M.; Inaba, Y.; Yamaguchi, I.; Endo, O.; Hammond, D.; Uchiyama, S.; Suzuki, G. Smoking topography and biomarkers of exposure among Japanese smokers: associations with cigarette emissions obtained using machine smoking protocols. Environ. Health Prev. Med. 2013, 18, 95-103, doi:10.1007/s12199-012-0293-7.

45. McClure, E.A.; Saladin, M.E.; Baker, N.L.; Carpenter, M.J.; Gray, K.M. Smoking topography and abstinence in adult female smokers. Addict. Behav. 2013, 38, 2833-2836, doi:10.1016/j.addbeh.2013.08.004.

46. Strasser, A.A.; Ashare, R.L.; Kaufman, M.; Tang, K.Z.; Mesaros, A.C.; Blair, I.A. The effect of menthol on cigarette smoking behaviors, biomarkers and subjective responses. Cancer Epidemiol. Biomarkers Prev. 2013, 22, 382-389, doi:10.1158/1055-9965.EPI-12-1097.

47. Tidey, J.W.; Rohsenow, D.J.; Kaplan, G.B.; Swift, R.M.; Ahnallen, C.G. Separate and combined effects of very low nicotine cigarettes and nicotine replacement in smokers with schizophrenia and controls. Nicotine Tob Res 2013, 15, 121-129, doi:10.1093/ntr/nts098.

48. Hammond, D.; O'Connor, R.J. Reduced nicotine cigarettes: smoking behavior and biomarkers of exposure among smokers not intending to quit. Cancer Epidemiol. Biomarkers Prev. 2014, 23, 2032-2040, doi:10.1158/1055-9965.Epi-13-0957.

49. Norton, K.J.; June, K.M.; O'Connor, R.J. Initial puffing behaviors and subjective responses differ between an electronic nicotine delivery system and traditional cigarettes. Tob. Induc. Dis. 2014, 12, 17, doi:10.1186/1617-9625-12-17.

50. Schneider, T.; De Jesus, S.; Prapavessis, H. The Effect of Acute Exercise on Smoking Topography: No Evidence for Cutting Down One Puff at a Time. Journal of Smoking Cessation 2014, 10, 146-153, doi:10.1017/jsc.2014.2.

51. Appleton, S.; Liu, J.; Lipowicz, P.J.; Sarkar, M. Effect of cigarette design on biomarkers of exposure, puffing topography and respiratory parameters. Inhal. Toxicol. 2015, 27, 174-180, doi:10.3109/08958378.2015.1021980.

52. Brinkman, M.C.; Kim, H.; Chuang, J.C.; Kroeger, R.R.; Deojay, D.; Clark, P.I.; Gordon, S.M. Comparison of True and Smoothed Puff Profile Replication on Smoking Behavior and Mainstream Smoke Emissions. Chem. Res. Toxicol. 2015, 28, 182-190, doi:10.1021/tx500318h.

53. Chung, S.; Kim, S.S.; Kini, N.; Fang, H.J.; Kalman, D.; Ziedonis, D.M. Smoking topography in Korean American and white men: preliminary findings. J Immigr Minor Health 2015, 17, 860-866, doi:10.1007/s10903-013-9921-6.

54. Gass, J.C.; Germeroth, L.J.; Wray, J.M.; Tiffany, S.T. The Reliability and Stability of Puff Topography Variables in Non-Daily Smokers Assessed in the Laboratory. Nicotine Tob Res 2016, 18, 484-490, doi:10.1093/ntr/ntv045.

55. Krebs, N.M.; Chen, A.; Zhu, J.; Sun, D.; Liao, J.; Stennett, A.L.; Muscat, J.E. Comparison of Puff Volume With Cigarettes per Day in Predicting Nicotine Uptake Among Daily Smokers. Am. J. Epidemiol. 2016, 184, 48-57, doi:10.1093/aje/kwv341.

56. Chen, A.; Krebs, N.M.; Zhu, J.; Muscat, J.E. Nicotine metabolite ratio predicts smoking topography: The Pennsylvania Adult Smoking Study. Drug Alcohol Depend. 2018, 190, 89-93, doi:10.1016/j.drugalcdep.2018.06.003.

57. Mercincavage, M.; Souprountchouk, V.; Tang, K.Z.; Dumont, R.L.; Wileyto, E.P.; Carmella, S.G.; Hecht, S.S.; Strasser, A.A. A Randomized Controlled Trial of Progressively Reduced Nicotine Content Cigarettes on Smoking Behaviors, Biomarkers of Exposure, and Subjective Ratings. Cancer Epidemiol. Biomarkers Prev. 2016, 25, 1125-1133, doi:10.1158/1055-9965.EPI-15-1088.

58. Mercincavage, M.; Lochbuehler, K.; Wileyto, E.P.; Benowitz, N.L.; Tyndale, R.F.; Lerman, C.; Strasser, A.A. Association of Reduced Nicotine Content Cigarettes With Smoking Behaviors and Biomarkers of Exposure Among Slow and Fast Nicotine Metabolizers: A Nonrandomized Clinical Trial. JAMA Netw Open 2018, 1, e181346-e181346, doi:10.1001/jamanetworkopen.2018.1346.

59. Pulcu, E. Self-report distortions of puffing topography in daily smokers. J. Health Psychol. 2016, 21, 1644-1654, doi:10.1177/1359105314560917.

60. Ross, K.C.; Dempsey, D.A.; St Helen, G.; Delucchi, K.; Benowitz, N.L. The Influence of Puff Characteristics, Nicotine Dependence, and Rate of Nicotine Metabolism on Daily Nicotine Exposure in African American Smokers. Cancer Epidemiol. Biomarkers Prev. 2016, 25, 936-943, doi:10.1158/1055-9965.EPI-15-1034.

61. Ross, K.C.; Juliano, L.M. Smoking Through a Topography Device Diminishes Some of the Acute Rewarding Effects of Smoking. Nicotine Tob Res 2016, 18, 564-571, doi:10.1093/ntr/ntv159.

62. Tidey, J.W.; Cassidy, R.N.; Miller, M.E. Smoking Topography Characteristics of Very Low Nicotine Content Cigarettes, With and Without Nicotine Replacement, in Smokers With Schizophrenia and Controls. Nicotine Tob Res 2016, 18, 1807-1812, doi:10.1093/ntr/ntw089.

63. Farris, S.G.; Aston, E.R.; Abrantes, A.M.; Zvolensky, M.J. Tobacco demand, delay discounting, and smoking topography among smokers with and without psychopathology. Drug Alcohol Depend. 2017, 179, 247-253, doi:10.1016/j.drugalcdep.2017.06.042.

64. Higgins, S.T.; Heil, S.H.; Sigmon, S.C.; Tidey, J.W.; Gaalema, D.E.; Stitzer, M.L.; Durand, H.; Bunn, J.Y.; Priest, J.S.; Arger, C.A., et al. Response to varying the nicotine content of cigarettes in vulnerable populations: an initial experimental examination of acute effects. Psychopharmacology 2017, 234, 89-98, doi:10.1007/s00213-016-4438-z.

65. Hsu, P.C.; Lan, R.S.; Brasky, T.M.; Marian, C.; Cheema, A.K.; Ressom, H.W.; Loffredo, C.A.; Pickworth, W.B.; Shields, P.G. Menthol Smokers: Metabolomic Profiling and Smoking Behavior. Cancer Epidemiol. Biomarkers Prev. 2017, 26, 51-60, doi:10.1158/1055-9965.EPI-16-0124.

66. Watson, C.V.; Richter, P.; de Castro, B.R.; Sosnoff, C.; Potts, J.; Clark, P.; McCraw, J.; Yan, X.; Chambers, D.; Watson, C. Smoking Behavior and Exposure: Results of a Menthol Cigarette Cross-over Study. Am. J. Health Behav. 2017, 41, 309-319, doi:10.5993/AJHB.41.3.10.

67. Bergeria, C.L.; Heil, S.H.; Bunn, J.Y.; Sigmon, S.C.; Higgins, S.T. Comparing Smoking Topography and Subjective Measures of Usual Brand Cigarettes Between Pregnant and Non-Pregnant Smokers. Nicotine Tob Res 2018, 20, 1243-1249, doi:10.1093/ntr/ntx148.

68. Kim, S.; Yu, S. Smoking Topography among Korean Smokers: Intensive Smoking Behavior with Larger Puff Volume and Shorter Interpuff Interval. Int. J. Environ. Res. Public Health 2018, 15, doi:10.3390/ijerph15051024.

69. Shiffman, S.; Scholl, S. Increases in Cigarette Consumption and Decreases in Smoking Intensity When Nondaily Smokers Are Provided With Free Cigarettes. Nicotine Tob Res 2018, 20, 1237-1242, doi:10.1093/ntr/ntx221.

70. Vansickel, A.R.; Edmiston, J.S.; Liang, Q.; Duhon, C.; Connell, C.; Bennett, D.; Sarkar, M. Characterization of puff topography of a prototype electronic cigarette in adult exclusive cigarette smokers and adult exclusive electronic cigarette users. Regul. Toxicol. Pharmacol. 2018, 98, 250-256, doi:10.1016/j.yrtph.2018.07.019.

71. Yuki, D.; Takeshige, Y.; Nakaya, K.; Futamura, Y. Assessment of the exposure to harmful and potentially harmful constituents in healthy Japanese smokers using a novel tobacco vapor product compared with conventional cigarettes and smoking abstinence. Regul. Toxicol. Pharmacol. 2018, 96, 127-134, doi:10.1016/j.yrtph.2018.05.001.

72. Watson, C.V.; Richter, P.; Li, Y.; Phillips, T.; Pickworth, W.B.; deCastro, R.B.; Potts, J.; Watson, C. Mouth Level Nicotine in a Clinical Setting versus Non-clinical Setting. Tobacco Regulatory Science 2019, 5, 229-241, doi:10.18001/trs.5.3.2.

73. ISO/TR 17219:2013 Review of human smoking behaviour and recommendations for a new ISO standard for the machine smoking of cigarettes; International Organisation for Standardisation: Geneva, Switzerland, 2013.
