# Supplementary material for: Characteristic Human Individual Puffing Profiles Can Generate More TNCO than ISO and Health Canada Regimes on Smoking Machine When the Same Brand Is Smoked
Source: Int J Environ Res Public Health. 2020 May 6;17(9):3225. doi: 10.3390/ijerph17093225 (PMC7246490; doi:10.3390/ijerph17093225)
Supplement: Supplementary file 1 [file ijerph-17-03225-s001.zip › Supp file Table S1.pdf]

International Journal of
Environmental Research and Public Health

Supplementary file A

Table S1: Literature reporting puffing parameters in chronological order since 2000.

Table S1: Literature reporting puffing parameters in chronological order since 2000

Author	Year	Sort	n	Gender	age	SD	No of cig/day	SD	Puff volume (ml)	SD	Puff duration (sec)	SD	Puff flow	SD	Puff interval (sec)	SD	Puff count	SD	Total volume (ml/cig)	SD	Total volume /day	Fagerström index	SD	Device	Comments
Buchhalter *et al* [1]	2000	own brand (9,5 mg tar, 0.73 mg nicotine)	10	♂/♀	23.2	3.4	15.3	4.1	38.4	11.7	1.2	0.4	*32.0*		35.0	17.9	10.3	2.1	*395.5*		*6.1*	4.0	1.2	CRSSPT	
Djordjevic *et al* [2]	2000	own brand (8.5 mg tar, 0.7 mg nicotine)	56	♂/♀	34.1	CI 31.3-37.2	14.9	CI 13.1-16.9	48.6	CI 45.2 - 25.3	1.5	CI 1.4-1.7	*32.4*		21.3	CI 19-23.8	12.7	CI 11.8-13.6	615.0	CI 566-668	9.5	3.3	CI 2.3-4.8	Tobacco Smoke Inhalation Testing System	
		own brand (15.4 mg tar, 1.11 mg nicotine)	77	♂/♀	34.7	CI 32.5-37.1	15.9	CI 14.3-17.8	44.1	CI 40.8-46.8	1.5	CI 1.4-1.6	*29.4*		18.5	CI 16.5-20.6	12.1	CI 11.3-12.9	523.0	CI 487-561	8.2	3.8	CI 2.7-5.3	Tobacco Smoke Inhalation Testing System	
Breland *et al* [3]	2002	own brand (10,2 mg tar, 0.8 mg nicotine)	20	♂/♀	21.6	SEM 1.1	18.9	SEM 0.7	49.8	SEM 3.3	1.7	SEM 0.1	*29.3*				8		*398.4*		*7.5*	5.6	SEM 0.3	Tobacco Smoke Inhalation Testing System	Fixed puff count
Breland *et al* [4]	2002	own brand (9,4 mg tar, 0.73 mg nicotine)	18	♂/♀	25.8	6.0	20.7	4.7	56.5	11.2	1.8	0.1	*31.4*		33.9	23.6	8		*452.0*		*9.4*	5.3	1.6	Tobacco Smoke Inhalation Testing System	Fixed puff count
Dixon *et al* [5]	2003	test cigarette (9.5 mg tar, 0.77 mg nicotine)	13						55.4	12.4	1.8	0.8	*31.7*		20.9	4.4	16.0	2.6	877.0	197.0				Orifice-type holder/flowmeter (design of Creighton *et al.* 1978)	
		test cigarette (8.8 mg tar, 0.48 mg nicotine)	13						56.7	11.6	1.8	0.8	*31.5*		20.8	3.6	16.1	3.0	890.0	152.0				Orifice-type holder/flowmeter (design of Creighton *et al.* 1978)	
		test cigarette (8.7 mg tar, 0.81 mg nicotine)	13						53.6	13.3	1.7	0.8	*31.7*		21.4	5.3	14.4	2.3	755.0	166.0				Orifice-type holder/flowmeter (design of Creighton *et al.* 1978)	
		test cigarette 8.1mg tar, 0.10 mg nicotine)	13						55.6	11.5	1.8	0.7	*31.4*		21.1	4.5	14.4	2.3	766.0	141.0				Orifice-type holder/flowmeter (design of Creighton *et al.* 1978)	
		test cigarette (7.7 mg tar, 0.22 mg nicotine)	13						55.8	13.0	1.8	0.8	*30.3*		20.0	3.7	15.6	3.3	870.0	136.0				Orifice-type holder/flowmeter (design of Creighton *et al.* 1978)	
Lee *et al* [6]	2003	own brand (16.7 mg tar, 1.2 mg nicotine)	7	♂/♀	37.0	25-44	25.0	10-38	30.8	SE 3.5	0.9	SE 0.1	44.6	SE 3.4	19.9	SE 3.5	*9.3*		*284.9*		*7.1*	6.8	4-10	CRSSPT	8, 8, 11, 10 puffs
		own brand (12.8 mg tar, 1.0 mg nicotine)	10	♂/♀	24.5	20-37	25.0	17-40	45.1	SE 4.9	1.2	SE 0.1	54.4	SE 4.8	23.2	SE 3.0	10.0		*451.0*		*11.3*	6.0	3-9	CRSSPT	
Aung *et al* [7]	2004	own brand	56	♂/♀	15.4	1.3	19.6	8.93	38.1	14.5	1.1	0.4	52.0	19.3	15.5	8.6	14.0	3.9	*534.0*		*10.5*	7.4	1.4	CRSSPT	Current non-marijuana-smoking teens
Dixon[$]	2004	10 mg tar, 1 mg nicotine	100	♂/♀					46.2		1.9		*24.2*		24.7		12.6		558.0						
		4,8 mg tar, 0.5 mg nicotine	100	♂/♀					58.6		1.9		*31.7*		21.7		16.6		939.0						
		2,8 mg tar, 0.3 mg nicotine	100	♂/♀					57.6		1.9		*30.6*		19.7		15.0		844.0						
		1,2 mg tar, 0.1 mg nicotine	100	♂/♀					63.4		1.9		*33.2*		18.7		18.8		1135.0						
Dixon[$]	2004	5,6 mg tar, 0.5 mg nicotine	50	♂/♀					52.8		1.7		*30.3*				12.5		644.0						
		1 mg tar, 0.1 mg nicotine	50	♂/♀					63.2		1.7		*36.3*				15.1		901.0						
Hughes *et al* [8]	2004	own brand (Light, ultralight, regular)	34	♂/♀	48.0		30.0	SEM 2	50.0	SEM 2							12.7	SEM 0.7	612.0	SEM 34	*18.4*	6.4	2.0	CRSSPT	

Table S1: Literature reporting puffing parameters in chronological order since 2000

Author	Year	Sort	n	Gender	age	SD	No of cig/day	SD	Puff volume (ml)	SD	Puff duration (sec)	SD	Puff flow	SD	Puff interval (sec)	SD	Puff count	SD	Total volume (ml/cig)	SD	Total volume /day	Fagerström index	SD	Device	Comments
		Omni light (12 mg tar, 0.8 mg nicotine) or Omni Full flavor King/100's (15 mg tar, 1.0 mg nicotine)	34	♂/♀	48.0		31.0	SEM 2	49.0	SEM 2							11.6	SEM 0.5	547.0	SEM 25	*17.0*	6.4	2.0	CRSSPT	
Lee *et al* [9]	2004	own brand (14.5 mg tar, 1.1 mg nicotine)	10	♂/♀	30.3	19-46	21.3	13-30	60.1	4.0	*1.1*		57.1	3.5			11.5	0.7	691.2		*14.7*	5.4	3-9	CRSSPT	
Strasser *et al* [10]	2004	own brand (mean 0.93mg nicotine)	113	♂/♀	45.8	10.7	21.2	7.8	54.8	16.9	1.6	0.5	34.3	8.2	24.8	11.9	12.7	4.0	675.7	244.7	*14.3*	5.5	2.1	CRSSPT	
Wood *et al* [11]	2004	own brand	24	♀	16.3	1.5	15.0	1-25	38.5	12.2	1.1	0.3	*35.0*		24.1	9.9	12.7	6.2	504.4	312.1	7.6	4.2	1.6	CRSSPT	
		own brand	25	♂	16.4	1.4	15.0	1-25	45.8	14.6	1.2	0.3	*38.2*		19.1	10.9	15.6	6.1	729.0	393.5	10.9	4.6	1.7	CRSSPT	
Zimmerman *et al* [12]	2004	own brand	92	♂/♀	15.2	1.5			38.2	12.5	1.1	0.4	51.1	16.0	16.8	10.9								CRSSPT	volunteers cessation trial NIDA
Hammond *et al* [13]	2005	own brand (10-14 mg tar)	58	♂/♀	37.1	11.1	19.3	8.0	54.2	10.3	1.5	0.2	38.1	5.6	33.5	17.6	11.3	3.4	612.0	195.7	*11.8*			CRSSPT	trial 1
		own brand (10-14 mg tar)	58	♂/♀	37.1	11.1	19.3	8.0	52.3	12.4	1.4	0.3	37.9	6.1	35.6	18.4	11.1	3.3	580.5	206.9	*11.2*			CRSSPT	trial 2
		Matinee Extra Mild (4 mg tar, 0.8 mg nicotine)	24	♂/♀	37.1	11.1	19.3	8.0	58.3	16.1							*13.4*		779.2	331.0	*15.0*			CRSSPT	trial 3
Hitsman *et al* [14]	2005	own brand (mean 0.8 mg nicotine)	8	♂/♀	35.5	16.9	18.8	2.3			2.4	0.7			36.8	17.2	10.8	3.6				5.6	1.7	Videotaping + handtiming	
Strasser *et al* [15]	2005	Marlboro Light 100 HP (10 mg tar, 0.8 mg nicotine)	12	♂/♀	21.6	4.5	17.5	12.5	63.4	SE 8.4	1.6	SE 0.2	42.5	SE 3.2	45		8		*507.2*		*8.9*			CReSS	Study 1, fixed puff count and IPI, unblocked
		Carlton 100 HP (1 mg tar, 0.1 mg nicotine)	12	♂/♀	21.6	4.5	18.0	12.5	85.1	SE 10.9	2.0	SE 0.3	44.5	SE 2.8	45		8		*680.8*		*12.3*			CReSS	Study 1, fixed puff count and IPI, unblocked
		Carlton 100 HP (1 mg tar, 0.1 mg nicotine)	12	♂	41.6	15.3	19.4	9.5	83.9		1.9		*62.2*		45		8		*671.5*		*13.0*	4.0	2.6	CReSS	Study 2, fixed puff count and IPI, unblocked, calculated mean (n=3)
Tidey *et al* [16]	2005	own brand (mean 1.1 mg nicotine)	20	♂/♀	51.0	12.0	29.0	11	47.5	10.4	1.3	0.2	*36.5*		42.0	21.5	8.9	2.3	429.0	159.0	*12.4*	7.4	1.3	CReSS	
Breland *et al* [17]	2006	own brand (9.89 mg tar, 0.74 mg nicotine)	35	♂/♀	22.4	CI 21.19-23.55	21.0	CI 3.46-5.55	51.0	CI 45.70-56.24	1.4	CI 1.24-1.52	*36.9*		30.7	CI 26.48-34.99	10.0	CI 8.85-11.21	*511.2*		*10.8*			CReSS	

Table S1: Literature reporting puffing parameters in chronological order since 2000

Author	Year	Sort	n	Gender	age	SD	No of cig/day	SD	Puff volume (ml)	SD	Puff duration (sec)	SD	Puff flow	SD	Puff interval (sec)	SD	Puff count	SD	Total volume (ml/cig)	SD	Total volume /day	Fagerström index	SD	Device	Comments
Franken *et al* [18]	2006	own brand	66	♂/♀	15.1	1.3	19.0	8.4	36.0	13.5	1.1	0.4	35.6	10.6	18.2	10.6	5		*180.0*		*3420.0*	7.2	1.3	CReSS	Fixed puff count
Hammond *et al* [19]	2006	own brand (9-15 mg tar)	51	♂/♀	37.1		>5		53.3	11.9	1.4	0.3	38.6	5.9	33.2	15.8	11.5	3.6	602.6	195.8	*3013.0*				Same data as Hammond et al [13]
		Matinee Extra Mild (4 mg tar, 0.4 mg nicotine)	21	♂/♀	37.1		>5		60.7	15.6	1.6	0.4	40.8	8.4	30.5	15.4	13.4	5.3	802.4	346.2	*4012.0*				Same data as Hammond et al [13]
Kassel *et al* [20]	2007	Research cigarette (15.9 mg tar, 1.14 mg nicotine)	16	♂/♀	17.5	1.1	3.4	1.2	45.4	22.2	1.1	0.4	57.2	18.9	24.2	12.9	15.1	6.3	713.1	461.8	*2.4*	2.2	1.4	CReSS	
		Research cigarette (17.9 mg tar, 0.06 mg nicotine)	19	♂/♀	17.5	1.1	3.8	2	40.1	18.7	1.0	0.4	53.4	19.0	23.0	13.2	23.2	8.9	988.8	547.2	*3.8*	2.1	1.4	CReSS	
Melikian *et al* [21]	2007	own brand (10.1 mg tar, 1.92 mg nicotine)	129	♀	33.1	CI 31.2-35	15.9	CI 14.7-17.1	37.6		1.3		*28.3*		20.4		13.5		507.8		*8.1*	4.1	CI 3.65-4.62	puff analyzer [22]	
		own brand (12.4 mg tar, 2.2 mg nicotine)	128	♂	35.0	CI 33.3-36.9	16.8	CI 15.5-18.2	45.8		1.5		*30.9*		20.7		12.0		553.0		*9.3*	4.6	CI 4.12-5.06	puff analyzer [22]	
Strasser *et al* [23]	2007	own brand (mean 12,9 mg tar, 0.94 mg nicotine)	50	♂/♀	44.5	12.1	21.3	8.1	60.5	CI 55.2-65.7	1.8	CI 1.6-1.9	35.5	CI 22.3-37.8	21.6	CI 19.1-24.1	14.3	CI 12.8-15.7	832.0	CI 737-926	*17.7*	5.5	1.9	CReSS	
		Quest (10 mg tar, 0.6 mg nicotine)	50	♂/♀	44.5	12.1	21.3	8.1	58.1	CI 53.3-62.8	1.8	CI 1.6-1.9	34.5	CI 32.0-37.0	21.6	CI 19.0-24.2	9.8	CI 9-10.8	540.3	CI 500-580	*11.5*	5.5	1.9	CReSS	
		Quest (10 mg tar, 0.3 mg nicotine)	50	♂/♀	44.5	12.1	21.3	8.1	55.9	CI 51.0-60.8	1.7	CI 1.5-1.8	34.4	CI 31.9-37.0	19.6	CI 16.9-22.3	9.9	CI 8.9-10.9	518.1	CI 478-558	*11.0*	5.5	1.9	CReSS	
		Quest (10 mg tar, 0.05 mg nicotine)	50	♂/♀	44.5	12.1	21.3	8.1	59.4	CI 54.6-64.3	1.8	CI 1.6-1.9	35.1	CI 32.5-37.8	18.6	CI 15.7-21.6	10.0	CI 9.1-10.9	570.5	CI 527-614	*12.2*	5.5	1.9	CReSS	
Strasser *et al* [24]	2007	own brand	119	♂/♀	45.8	10.8	21.2	8.1	55.1	16.9							12.7	4.0	669.6	243.1	*14.2*	5.5	2.1	CReSS	
Attwood *et al* [25]	2008	own brand	55	♂/♀	22.0	4.0	11.0	5	49.0	12.0	1.6	0.4	*31.6*		14.0	5.0	17.0	6.0	*833.0*		*9.2*	4.0	2.0	CReSS	
Collins *et al* [26]	2008	own brand	67	♂/♀	15.3	1.3	>10		38.7		1.1		48.1				6		*232.1*		*2.3*	>5		CReSS	Fixed puff count
Rees *et al* [27]	2008	Marlboro Light (11 mg tar, 0.8 mg nicotine)	17	♂/♀	27.7	CI 24.3-31.0	15.7	CI 12.0-19.3	50.7	19.6	1.4	0.5	*37.6*		32.1	11.9	11.4	3.0	*578.0*		*9.1*	3.9	CI 2.5-5.4	CReSS	Supervised - Tampa group
		Marlboro Light (11 mg tar, 0.8 mg nicotine)	17	♂/♀	27.7	CI 24.3-31.0	15.7	CI 12.0-19.3	51.2	17.3	1.4	0.5	*35.8*		37.3	22.1	11.9	4.0	*609.3*		*9.6*	3.9	CI 2.5-5.4	CReSS	Unsupervised - Tampa group

Table S1: Literature reporting puffing parameters in chronological order since 2000

Author	Year	Sort	n	Gender	age	SD	No of cig/day	SD	Puff volume (ml)	SD	Puff duration (sec)	SD	Puff flow	SD	Puff interval (sec)	SD	Puff count	SD	Total volume (ml/cig)	SD	Total volume /day	Fagerström index	SD	Device	Comments
		Marlboro Light (11 mg tar, 0.8 mg nicotine)	15	♂/♀	27.8	CI 35.5-32.1	15.9	CI 9.5-22.2	47.4	16.9	1.3	0.4	*36.7*		24.8	11.5	13.1	4.8	*620.9*		*9.9*	4.4	CI 3.0-5.8	CReSS	Supervised - Salt Lake City group
		Marlboro Light (11 mg tar, 0.8 mg nicotine)	15	♂/♀	27.8	CI 35.5-32.1	15.9	CI 9.5-22.2	41.0	18.0	1.3	0.5	*32.3*		26.8	10.4	11.9	4.7	*487.9*		*7.8*	4.4	CI 3.0-5.8	CReSS	Unsupervised - Salt Lake City group
		Marlboro ultraLight (6 mg tar, 0.5 mg nicotine)	17	♂/♀	27.7	CI 24.3-31.0	15.7	CI 12.0-19.3	51.4	19.0	1.3	0.4	*38.9*		33.0	14.9	11.3	2.6	*580.8*		*9.1*	3.9	CI 2.5-5.4	CReSS	Supervised - Tampa group
		Marlboro ultraLight (6 mg tar, 0.5 mg nicotine)	17	♂/♀	27.7	CI 24.3-31.0	15.7	CI 12.0-19.3	49.9	18.3	1.4	0.5	*35.6*		35.1	15.3	10.5	2.6	*524.0*		*8.2*	3.9	CI 2.5-5.4	CReSS	Unsupervised - Tampa group
		Marlboro ultraLight (6 mg tar, 0.5 mg nicotine)	15	♂/♀	27.8	CI 35.5-32.1	15.9	CI 9.5-22.2	50.1	15.6	1.4	0.4	*36.3*		21.8	10.4	13.9	5.6	*696.4*		*11.1*	4.4	CI 3.0-5.8	CReSS	Supervised - Salt Lake City group
		Marlboro ultraLight (6 mg tar, 0.5 mg nicotine)	15	♂/♀	27.8	CI 35.5-32.1	15.9	CI 9.5-22.2	52.2	15.7	1.5	0.5	*34.8*		24.7	12.3	12.1	4.6	*631.6*		*10.0*	4.4	CI 3.0-5.8	CReSS	Unsupervised - Salt Lake City group
		Marlboro UltraSmooth (5 mg tar, 0.4 mg nicotine)	17	♂/♀	27.7	CI 24.3-31.0	15.7	CI 12.0-19.3	54.2	19.4	1.4	0.4	*39.0*		28.4	8.4	10.2	2.9	*552.8*		*8.7*	3.9	CI 2.5-5.4	CReSS	Activated carbon in filter - Supervised - Tampa group
		Marlboro UltraSmooth (5 mg tar, 0.4 mg nicotine)	17	♂/♀	27.7	CI 24.3-31.0	15.7	CI 12.0-19.3	53.5	19.2	1.4	0.5	*37.4*		33.6	15.8	9.3	2.4	*497.6*		*7.8*	3.9	CI 2.5-5.4	CReSS	Activated carbon in filter - Unsupervised - Tampa group
		Marlboro UltraSmooth (5 mg tar, 0.4 mg nicotine)	15	♂/♀	27.8	CI 35.5-32.1	15.9	CI 9.5-22.2	56.7	15.2	1.5	0.4	*38.6*		23.9	9.2	12.5	5.0	*708.8*		*11.3*	4.4	CI 3.0-5.8	CReSS	Activated carbon in filter - Supervised - Salt Lake City group
		Marlboro UltraSmooth (5 mg tar, 0.4 mg nicotine)	15	♂/♀	27.8	CI 35.5-32.1	15.9	CI 9.5-22.2	54.6	19.4	1.5	0.5	*35.9*		24.6	12.2	11.7	5.2	*638.8*		*10.2*	4.4	CI 3.0-5.8	CReSS	Activated carbon in filter - Unsupervised - Salt Lake City group
Shahab *et al* [28]	2008	own brand	131	♂/♀	31.4	10.4	13.4	5.7	54.2	14.0	1.5	0.5	37.7	7.8	25.8	10.3	13.6	4.5	712.0	232.0	*9.5*	2.3	1.4	CReSS	
Blank *et al* [29]	2009	own brand (mean 14.9 mg tar, 1.1 mg nicotine)	30	♂/♀	32.3	11.0	19.6	4.7	44.4	13.6	1.7	0.6	*26.1*		18.2	7.6	9.4	3.0	411.4	170.7	*8.1*	6.0	2.0	Portable CReSS	
		own brand (mean 14.9 mg tar, 1.1 mg nicotine)	30	♂/♀	32.3	11.0	19.6	4.7	57.0	20.1	1.7	0.7	*33.5*		18.1	8.8	9.7	3.3	578.7	327.2	*8.1*	6.0	2.0	Desktop CReSS	
		Merit ultra-light (5 mg tar, 0.5 mg nicotine)	30	♂/♀	32.3	11.0	19.6	4.7	52.8	12.6	1.9	0.6	*27.8*		16.7	7.7	10.1	3.4	528.5	207.2	*10.4*	6.0	2.0	Portable CReSS	
		Merit ultra-light (5 mg tar, 0.5 mg nicotine)	30	♂/♀	32.3	11.0	19.6	4.7	60.3	20.0	2.0	0.8	*30.2*		15.2	7.2	10.2	3.1	614.2	301.1	*8.1*	6.0	2.0	Desktop CReSS	
Dickens *et al* [30]	2009	Research cigarette (Virginia tobacco, 8.8 mg nicotine)	1	♂/♀					95.0	2.0	3.3	0.2	29.5	2.4					572.0					Smoker Analyser (SA7)	

Table S1: Literature reporting puffing parameters in chronological order since 2000

Author	Year	Sort	n	Gender	age	SD	No of cig/day	SD	Puff volume (ml)	SD	Puff duration (sec)	SD	Puff flow	SD	Puff interval (sec)	SD	Puff count	SD	Total volume (ml/cig)	SD	Total volume /day	Fagerström index	SD	Device	Comments
			1	♂/♀					72.0	1.0	1.7	0.3	40.7	1.7					573.0					Smoker Analyser (SA7)	
			1	♂/♀					63.0	13.0	1.9	0.3	35.0	2.9					376.0					Smoker Analyser (SA7)	
			1	♂/♀					57.0	1.0	1.7	0.1	34.5	1.1					400.0					Smoker Analyser (SA7)	
			1	♂/♀					119.0	7.0	2.6	0.5	27.5	3.0					717.0					Smoker Analyser (SA7)	
			1	♂/♀					73.0	4.0	1.5	0.2	35.0	0.9					440.0					Smoker Analyser (SA7)	
			1	♂/♀					55.0	5.0	3.3	0.2	29.5	2.4					441.0					Smoker Analyser (SA7)	
Grainge *et al* [28]	2009	regular	130	♂/♀	31.4	10.5	13.6	6.1	56.2	13.8	1.6	0.5	*35.6*		26.6	10.5	13.2	4.1	711.7	208.3	*9.7*			Portable CReSS	
Moolchan *et al* [31]	2009	own brand	85	♂/♀	15.3	1.2	18.6	8.5	38.5	15.6							5		599.5	201.6	*11.1*	7.0	1.2	CReSS	Fixed puff count
Collins *et al* [32]	2010	own brand	89	♂/♀	15.3	1.3	18.6	9.2	41.5	1.2	1.3	0.0	53.9	2.0	14.5	0.7	15.2		*630.8*		*11.7*	7.3	1.3	CReSS	
O'Connor *et al* [33]	2010	own brand (Newport, Marlboro, Camel)	83	♂/♀	39.6	9.9	17.0	8.8	57.7	CI 51.8-63.2	1.8	CI 1.57-1.97	36.5	CI 33.4-39.7	17.6	CI 14.8-20.5	14.4	CI 13.1-15.7	828.0	CI 750-905	*14.1*			Portable CReSS	Same data as June et al [34]
		own brand (Newport, Marlboro, Camel)	77	♂/♀	49.0	11.9	17.9	9.3	56.7	Ci 51.2-62.1	1.9	CI 1.70-2.08	31.5	CI 28.5-34.6	21.3	CI 18.6-24.0	13.7	CI 12.5-15.0	611.0	CI 539-682	*10.9*			Portable CReSS	Same data as June *et al* [34], Reduced Ignition Propensity type
June *et al* [35]	2011	Own brand (Number 7, Peter Jackson, Players, DuMaurier)	42	♂/♀	32.6	12.3	18.0	7.1	59.6	CI 54.2-64.9	1.7	CI 1.6-1.9	36.8	CI 33.8-39.8	19.2	CI 17.0-21.4	14.1	CI 12.7-15.5	834.9	CI 733.1-936.7	*15.0*			Portable CReSS	
		Own brand (Number 7, Peter Jackson, Players, DuMaurier)	42	♂/♀	32.6	12.3	18.0	7.1	60.3	CI 54.9-65.8	1.7	CI 1.6-1.9	37.2	CI 34.9-39.6	17.9	CI 15.1-20.6	14.2	CI 12.6-15.9	831.7	CI 745.8-917.6	*15.0*			Portable CReSS	Reduced Ignition Propensity type
McKee *et al* [36]	2011	own brand	37	♂/♀	37.7	10.6	18.4	7.96							32.9		9.7					6.1	2.3	Portable CReSS	
Strasser *et al* [37]	2011	own brand	109	♂/♀	45.5	10.8	20.5	8.4											785.1	284.9	*16.1*	4.9	2.1	Portable CReSS	
Veilleux *et al* [38]	2011	own brand	78	♂/♀	15.7	0.6	*3.29*		35.2	16.2	1.1	0.4	43.6	15.6	19.2	8.9	16.5	6.1	582.7		*1.9*	2.7	1.4	Portable CReSS	23.06 cig/week

Table S1: Literature reporting puffing parameters in chronological order since 2000

Author	Year	Sort	n	Gender	age	SD	No of cig/day	SD	Puff volume (ml)	SD	Puff duration (sec)	SD	Puff flow	SD	Puff interval (sec)	SD	Puff count	SD	Total volume (ml/cig)	SD	Total volume /day	Fagerström index	SD	Device	Comments
Williams *et al* [39]	2011	own brand	86	♂/♀	38.0	12.0	20.0	7.7	46.2	SE 0.15	1.3	SE 0.01	37.1	SE 0.1	21.0	SE 0.14	12.3	SE0.15	540.5	SE7.01	*10.8*	5.5	1.9	Portable CReSS	control group, same data as Williams *et al* [40]
Brinkman *et al* [41]	2012	Kent 100 (non-menthol)	9	♂/♀	23.0	3.5	16.9	SEM 0.7	53.9	SEM 1.0	2.2	SEM 0.1	28.1	SEM 0.6	30.6	SEM 0.6	10.8	SEM 0.5	563.0	SEM20	*9.5*			Sodim SPA-D Smoking Puff Analyzer	
Faulkner *et al* [42]	2012	own brand	18	♂/♀	24.6	5.9	15.2	4.6	54.7	16.7	1.8	0.6	*30.8*		11.9	5.1	16.5	6.3	*903.2*		*13.7*	4.5	2.3	Portable CReSS	
		Benson & Hedges Light 100 (menthol)	9	♂/♀	23.0	3.5	15.2	SEM 0.8	54.7	SEM 0.8	2.4	SEM 0.1	25.6	SEM 0.5	25.6	SEM 0.5	11.6	SEM 0.5	636.0	SEM 27	*9.7*			Sodim SPA-D Smoking Puff Analyzer	
Perkins *et al* [43]	2012	own brand	94	♂/♀	26.8	SE 1	19.5	SE 0.6									12.6		568.7		*11.1*	4.7	SE 0.2	Portable CReSS	
Matsumoto *et al* [44]	2013	Top 10 brands Japan	101	♂/♀	40.0	11.0	18.4	7.5	54.3	14.1							14.5	3.6	767.2	259.5	14.5			Portable CReSS	
		Pianissimo, Mild Seven One (1 mg tar, 0.1 mg nicotine)	14	♂/♀	39.2	10.2	18.7	8.3	64.6	12.3							17.9	3.2	1160.1	302.7	22.6			Portable CReSS	
		Mild Seven One Extra Lights, Caster Mild, Mild Seven Super Lights (3-6 mg tar, 0.3-0.5 mg nicotine)	38	♂/♀	40.1	12.6	17.8	7.2	56.0	12.2							15.0	3.1	810.0	179.6	14.4			Portable CReSS	
		Cabin Mild, Mild Seven Lights, Mild Seven Original (8-10 mg tar, 0.6-0.8 mg nicotine)	27	♂/♀	41.9	11.2	18.2	6.6	46.9	12.3							13.4	3.4	609.4	158.7	11.4			Portable CReSS	
		Hope, Seven Stars (14 mg tar, 1.1-1.2 mg nicotine)	22	♂/♀	38.0	8.4	19.3	8.8	53.9	15.8							12.6	3.1	638.7	136.9	13.0			Portable CReSS	
McClure *et al* [45]	2013	own brand	135	♀	31.9	7.6	16.2	7	49.9	15.8	1.6	0.5	32.7	9.1			11.7	3.9	*583.8*		*9.5*	4.9	2.2	Portable CReSS	
Strasser *et al* [46]	2013	own brand	78	♂/♀	38.4	11.7	18.0	8	*54.7*		1.7	0.1	*32.2*		30.1	2.1	11.2	0.6	612.6	74.6	*11.0*	5.8	1.8	Portable CReSS	Day 1 - cigarette 1
		own brand	78	♂/♀	38.4	11.7	18.0	8	*46.7*		1.7	0.1	*27.4*		33.7	3.2	11.1	0.8	517.9	36.8	*9.3*	5.8	1.8	Portable CReSS	Day 1 - cigarette 2
		own brand	78	♂/♀	38.4	11.7	18.0	8	*44.5*		1.6	0.1	*27.8*		31.4	3.0	12.6	0.7	560.7	42.7	*10.1*	5.8	1.8	Portable CReSS	Day 5
Tidey *et al* [47]	2013	own brand	26	♂/♀	44.5	10.9	23.5	6.5											1451.0	835.0	*34.1*	6.7	1.8	Desktop CReSS	

Table S1: Literature reporting puffing parameters in chronological order since 2000

Author	Year	Sort	n	Gender	age	SD	No of cig/day	SD	Puff volume (ml)	SD	Puff duration (sec)	SD	Puff flow	SD	Puff interval (sec)	SD	Puff count	SD	Total volume (ml/cig)	SD	Total volume /day	Fagerström index	SD	Device	Comments
Hammond *et al* [48]	2014	own brand	72	♂/♀	37.2	11.9	16.1	6.3	63.5	22.4							16.1	6.3	977.9	420.8	*15.7*	4.9	2.3	Portable CReSS	
		Quest 1 (0.6 mg nicotine)	71	♂/♀	37.2	11.9	14.5	4.6	64.3	22.3							14.5	4.6	910.1	520.3	*13.2*	4.8	2.2	Portable CReSS	
		Quest 2 (0.3 mg nicotine)	71	♂/♀	37.2	11.9	13.8	4.3	64.2	17.2							13.8	4.3	834.2	190.4	*11.5*	4.8	2.1	Portable CReSS	
		Quest 3 (0.05 mg nicotine)	72	♂/♀	37.2	11.9	13.2	4.5	69.3	18.8							13.2	4.5	890.7	353.6	*11.8*	4.4	2.3	Portable CReSS	
Norton *et al* [49]	2014	own brand	16	♂/♀	45.5	SE 3.5	16.1	SE 1.4	67.5	SE 6.3	3.0	SE 1	36.1	SE 1.8	21.3	SE 6.2	13.2	SE 1.1	829.0	SE 51.4	*13.3*			Portable CReSS	
Schneider *et al* [50]	2014	own brand	25	♂/♀	45.9	12.4	16.7	4.6	62.2	18.5	1.7	0.5	*36.8*		18.7	7.7	10.2	2.7	*635.5*		*10.6*	5.6	2.0	Portable CReSS	
		own brand	25	♂/♀	45.9	12.4	16.7	4.6	58.8	18.3	1.6	0.4	*36.3*		19.2	7.6	11.4	2.7	*667.5*		*11.2*	5.6	2.0	Portable CReSS	
		own brand	23	♂/♀	39.1	13.8	15.6	4.22	55.3	18.5	1.7	0.4	*31.8*		14.3	5.0	14.2	4.7	*783.6*		*12.2*	4.2	1.9	Portable CReSS	
		own brand	23	♂/♀	39.1	13.8	15.6	4.22	54.8	16.9	1.7	0.5	*32.2*		15.0	5.5	13.9	5.6	*763.9*		*11.9*	4.2	1.9	Portable CReSS	after 10 minutes exercise
Appleton *et al* [51]	2015	own brand (>13 mg tar)	20	♂	35.3	6.1	10-30		69.7	18.6	2.3	0.6	*30.3*		38.6	10.8	9.8	2.3	*683.1*		*6.8*			Portable CReSS	Total volume if 10 cig/day
		Test cigarette High Tar (~15 mg tar)	20	♂	35.4	6.4	22.4-24.0		59.4	18.3	2.0	0.6	*29.4*		37.2	10.2	8.9	2.2	*528.4*		*11.8*			Portable CReSS	Total volume if 22.4 cig/day
		Test Cigarette Low tar (~7 mg tar)	19	♂	35.1	6.2	20.5-22.6		69.3	19.2	2.4	0.7	*29.5*		33.3	9.0	9.5	2.5	*658.4*		*13.5*			Portable CReSS	Total volume if 20.5 cig/day
Brinkman *et al* [52]	2015	GPC Full flavor and GPC Ultra Light	24	♂/♀	18-30		>20		55.6	CI 53.71-57.49	2.1	CI 2.042-2.198	27.2	CI 26.22-28.21	19.3	CI 18.48-20.14	*11.6*		643.8	CI 627.2-660.9	*12.9*			Sodim SPA-D Smoking Puff Analyzer	Based on puff profiles of Brinkman *et al* [41]
		GPC Full flavor (15.2 mg tar, 0.97 mg nicotine)	24	♂/♀	18-30		>20		51.9	CI 49.42-54.45	2.0	CI 1.9-2.13	26.7	CI 25.40-28.04	21.0	CI 19.87-22.20	*11.5*		598.5	CI 579.84-617.77	*12.0*			Sodim SPA-D Smoking Puff Analyzer	Based on puff profiles of Brinkman *et al* [41]
		GPC Ultra Light (5.8 mg tar, 0.51 mg nicotine)	24	♂/♀	18-30		>20		59.6	CI 56.94-62.37	2.2	CI 2.14-2.34	27.7	CI 26.27-29.27	17.7	CI 16.59-18.86	*11.6*		693.4	CI 666.79-721.06	*13.9*			Sodim SPA-D Smoking Puff Analyzer	Based on puff profiles of Brinkman *et al* [41]
Chung *et al* [53]	2015	own brand - white	26	♂	39.2	10.1	>12		57.2	SE 2.69	1.6	SE 0.06	37.1	SE 1.70	25.0	SE 1.52	12.3	SE 0.61	*700.8*		*8.4*	5.8	2.1	Portable CReSS	
		own brand- korean	27	♂	37.3	9.7	12.0	1.82	59.6	SE 3	1.5	SE 0.07	42.1	SE 1.94	13.5	SE 1.7	13.8	SE 0.69	*820.6*		*9.8*	4.6	2.1	Portable CReSS	
Gass *et al* [54]	2016	own brand	107	♂/♀	25.0	5.9	2.7	2.2	31.4	13.0	1.2	0.5	26.8	7.9	28.8	45.4	13.5	4.5	413.6	182.2	*1.1*	1.1	1.5	Portable CReSS	
Krebs *et al* [55]	2016	own brand	332	♂/♀	37.6	11.6	16.5	6.2	48.2	14.9	1.6	0.4	34.0	1.3	26.6	23.8	12.2	4.8	*588.0*		5526.6	4.4	2.3	SPA-M	Pennsylvania Adult Smoking Study, same

Table S1: Literature reporting puffing parameters in chronological order since 2000

Author	Year	Sort	n	Gender	age	SD	No of cig/day	SD	Puff volume (ml)	SD	Puff duration (sec)	SD	Puff flow	SD	Puff interval (sec)	SD	Puff count	SD	Total volume (ml/cig)	SD	Total volume /day	Fagerström index	SD	Device	Comments
																									study as Chen *et al* [56]
Mercincagave *et al* [57]	2016	Own brand	63	♂/♀	40.7	12.7	19.3	CI 17.68-20.98	56.5	CI 52.45-60.47	1.8	CI 1.63-1.89	*32.1*		24.4	21.34-27.40	13.8	CI 12.63-14.88	756.0	CI 690.68-821.34	*14.6*	5.6	1.9	Portable CReSS	same study as Mercincagave *et al* [58]
		Quest 1 (0.6 mg nicotine)	63	♂/♀	40.7	12.7	22.6	CI 20.93-24.36	52.2	CI 48.13-56.30	1.7	CI 1.53-1.79	*31.5*		20.9	CI 18.36-23.46	10.8	CI 9.87-11.81	541.0	CI 489.42-592.51	*12.2*	5.6	1.9	Portable CReSS	same study as Mercincagave *et al* [58]
		Quest 2 (0.3 mg nicotine)	63	♂/♀	40.7	12.7	24.2	CI 22.35-26.01	52.7	CI 48.91-56.37	1.8	CI 1.61-1.88	*30.1*		19.1	CI 16.51-21.58	10.9	CI 9.92-11.91	545.1	CI 499.34-590.86	*13.2*	5.6	1.9	Portable CReSS	same study as Mercincagave *et al* [58]
		Quest 3 (0.05 mg nicotine)	63	♂/♀	40.7	12.7	20.6	CI 18.53-22.58	61.1	CI 57.18-65.08	1.9	CI 1.79-2.05	*31.8*		17.5	CI 15.04-19.91	11.8	CI 10.63-12.88	691.1	CI 632.34-749.91	*14.2*	5.6	1.9	Portable CReSS	same study as Mercincagave *et al* [58]
Pulcu *et al* [59]	2016	own brand	110	♂/♀	28.2	9.4	15.8	6.2							26.6	11.4	14.4	4.0						Portable CReSS	
Ross *et al* [60]	2016	own brand	59	♂/♀	34.9	10.1	14.0	6.4	45.9	16.6	1.5	0.1	31.3	8.4	21.4	9.4	12.4	3.9	536.4	117.0	*7.5*	5.1	2.0	Portable CReSS	first cigarette of the day
			59	♂/♀	34.9	10.1	14.0	6.4	46.6	16.0	1.5	0.5	32.1	8.8	19.4	8.4	12.9	4.0	554.5	164.7	*7.8*	5.1	2.0	Portable CReSS	after 4 hours ad libitum smoking
			30	♂	35.5	11.1	14.6	6.9	50.9	17.7	1.7	0.5	31.4	10.5	21.2	7.3	11.4	2.8	559.5	200.9	*8.2*	4.5	2.0	Portable CReSS	first cigarette of the day
			30	♂	35.5	11.1	14.6	6.9	52.2	17.6	1.7	0.5	31.9	11.0	18.7	8.4	12.2	3.2	582.2	171.8	*8.5*	4.5	2.0	Portable CReSS	after 4 hours ad libitum smoking
			29	♀	34.4	9.2	13.4	5.9	40.7	13.7	1.4	0.4	31.1	5.8	21.6	11.3	13.4	4.6	512.5	148.1	*6.9*	5.7	1.8	Portable CReSS	first cigarette of the day
			29	♀	34.4	9.2	13.4	5.9	40.9	12.0	1.3	0.4	32.2	5.9	20.0	8.4	13.7	4.7	526.7	155.3	*7.1*	5.7	1.8	Portable CReSS	after 4 hours ad libitum smoking
Ross *et al* [61]	2016	own brand (non-menthol)	58	♂/♀	40.0	13.2	11.8	5.62	*82.9*		2.1	0.5	39.9	8.3			14.6	4.6	*1213.3*		*14.4*	4.0	2.3	Portable CReSS	
Tidey *et al* [62]	2016	own brand	23	♂/♀	45.0	11.1	23.3	6.2	44.0		1.4		*31.4*		35.0		11.0		500.0		*11.7*			Portable CReSS	Graph data, originates from Tidey *et al* [47]
Farris *et al* [63]	2017	own brand	64	♂/♀	44.3	9.5	14.8	5.47	63.2	24.4	1.8	0.6	*35.9*		14.3	5.4	20.4	7.4	*1290.1*		*19.1*	4.6	1.5	Portable CReSS	
Higgins *et al* [64]	2017	SPECTRUM 15.8 mg nicotine/g tobacco	26	♂/♀	36.7	10.8	17.5	10.15	38.9	15.1	1.4	0.4	*28.8*		19.9	9.3	15.0	8.3	463.8	211.9	*8.1*	5.2	2.7	Desktop CReSS	
		SPECTRUM 5.2 mg nicotine/g tobacco	26	♂/♀	36.7	10.8	17.5	10.16	35.6	16.1	1.2	0.5	*28.9*		27.8	24.2	12.0	5.0	370.7	212.2	*6.5*	5.2	2.7	Desktop CReSS	
		SPECTRUM 2.4 mg nicotine/g tobacco	26	♂/♀	36.7	10.8	17.5	10.17	41.5	12.6	1.4	0.4	*29.9*		21.0	8.2	11.3	3.2	444.4	200.1	*7.8*	5.2	2.7	Desktop CReSS	
		SPECTRUM 0.4 mg nicotine/g tobacco	26	♂/♀	36.7	10.8	17.5	10.18	38.6	12.5	1.4	0.4	*28.0*		21.3	6.1	11.7	3.1	411.9	184.2	*7.2*	5.2	2.7	Desktop CReSS	
Hsu *et al* [65]	2017	own brand (non-menthol)	34	♂/♀	41.4	10.9	>10		52.7	18.0	1.6	0.8	*32.9*		21.0	14.7	12.3	6.6	690.3	547.8	*6.9*	5.4	2.7	CReSS	
		own brand (menthol)	71	♂/♀	43.8	9.3	>10		55.7	18.5	1.8	0.6	*30.9*		20.0	12.7	12.6	7.5	691.7	455.9	*6.9*	4.8	2.1	CReSS	

Table S1: Literature reporting puffing parameters in chronological order since 2000

Author	Year	Sort	n	Gender	age	SD	No of cig/day	SD	Puff volume (ml)	SD	Puff duration (sec)	SD	Puff flow	SD	Puff interval (sec)	SD	Puff count	SD	Total volume (ml/cig)	SD	Total volume /day	Fagerström index	SD	Device	Comments
Watson *et al* [66]	2017	Kent 100 (non-menthol)	42	♂/♀	35.0		23.0		47.7	SEM 1.5	1.2	SEM 0.03	*40.8*				14.4	SEM 0.5	*686.9*		*15.8*			Portable CReSS	
		Benson & Hedges Light 100 (menthol)	42	♂/♀	35.0		22.0		52.2	SEM 2.7	1.2	SEM 0.03	*42.4*				15.1	SEM 0.45	*788.2*		*17.3*			Portable CReSS	
Bergeria *et al* [67]	2018	own brand	89	♀	29.7	6.4	15.3	6.1	48.3	2.9	1.3	0.1	36.5	1.4	20.5	1.0	14.8	0.6	*716.4*		*11.0*	4.6	2.2	Desktop CReSS	
		own brand	20	♀	30.3	4.9	12.6	5.4	48.2	5.0	1.3	0.1	35.1	2.4	23.7	2.6	14.0	1.4	*674.9*		*8.5*	4.4	2.2	Desktop CReSS	pregnant
Chen *et al* [56]	2018	own brand	326	♂/♀	37.6	11.6	16.5	8.1	48.3	14.9	1.6	0.4	*30.2*				7.6	4.6	360.0	222.0	5.5	4.4	2.3	SPA-M	Pennsylvania Adult Smoking Study, same data as Krebs *et al* [55]
Kim *et al* [68]	2018	own brand	300	♂/♀	~30		0->20		median 60.6	IQR 49.9-73.9	median 1.5528	IQR 1.243-1.923	median 40.4	IQR 32.1-51.1	median 8.8289	IQR 6.5286-1.11961	median 15.5	IQR 13.5-19.0	*969.6*					Portable CReSS	IQR interquartile range
			250	♂	~30		0->20		median 32.7	IQR 52.7-75.5	median 1.6	1.2-1.9	median 41.2	IQR 33.1-52.2	median 8.9	IQR 6.5-11.2	median 15.5	IQR 13-19	*971.9*					Portable CReSS	
			50	♀	~30		0->20		median 53.5	IQR 42-64.2	median 1.4	1.2-1.8	median 36.4	IQR 30-43.7	median 8.3	IQR 6.2-11	median 17.5	IQR 15-21	*936.3*					Portable CReSS	
Mercincagave *et al* [58]	2018	own brand (non menthol)	84	♂/♀	43.0	12.1	13.9	CI 12.5-15.2	52.7	CI 48.1-57.4	1.7	CI 1.6-1.8	*31.0*		26.1	CI 23.2-28.9	14.7	CI 13.6-15.9	744.0	CI 681-806	*10.3*	5.5	1.8	CReSS	
		SPECTRUM 400 (9 mg tar, 0.26 mg nicotine)	84	♂/♀	43.0	12.1	16.5	CI 14.9-18.1	49.4	CI 44.4-54.3	1.7	CI 1.5-1.8	*29.1*		22.3	CI 19.7-24.8	11.2	CI 10.4-11.9	537.0	CI 479-595	*8.9*	5.5	1.8	CReSS	Research cigarette with reduced nicotine content (5.2mg nicotine/g tobacco)
		SPECTRUM 200 (9 mg tar, 0.07 mg nicotine)	84	♂/♀	43.0	12.1	14.8	CI 13.2-16.5	50.1	CI 45.5-54.6	1.8	CI 1.7-2.0	*27.8*		20.7	CI 18.2-23.2	12.3	CI 11.5-13.1	598.0	CI 547-649	*8.9*	5.5	1.8	CReSS	Research cigarette with reduced nicotine content (1mg nicotine/g tobacco)
Shiffman *et al* [69]	2018	own brand	190	♂/♀	38.0	13.8	2.0		*39.6*		*1.5*		*25.7*				15.8	6.0	625.6	209.8	*1.2*			Portable CReSS	bought, total puff time known
		own brand	190	♂/♀	38.0	13.8	3.3		*38.4*		*1.5*		*25.7*				15.1	6.1	581.2	227.6	*1.9*			Portable CReSS	provided for free, total puff time known
Vansickel *et al* [70]	2018	own brand	13	♂/♀	45.0	11.0	15.4	4	44.1	10.5	1.7	0.4	28.6	6.1										Sodim SPA-D Smoking Puff Analyzer	

Table S1: Literature reporting puffing parameters in chronological order since 2000

Author	Year	Sort	n	Gender	age	SD	No of cig/day	SD	Puff volume (ml)	SD	Puff duration (sec)	SD	Puff flow	SD	Puff interval (sec)	SD	Puff count	SD	Total volume (ml/cig)	SD	Total volume /day	Fagerström index	SD	Device	Comments
Yuki *et al* [71]	2018	own brand (~6.5 mg tar)	20	♂/♀	30.9	12.5	17.3	6.6	58.5	20.2	1.6	0.4	*36.1*				14.6	4.2	858.0	369.0	*14.8*	4.3	1.7	Portable CReSS	Baseline
		own brand (~6.5 mg tar)	20	♂/♀	30.9	12.5	17.3	6.6	58.8	19.0	1.8	0.7	*32.3*				14.6	3.8	857.0	296.0	*14.8*	4.3	1.7	Portable CReSS	day 1
		own brand (~6.5 mg tar)	20	♂/♀	30.9	12.5	17.3	6.6	62.8	19.2	1.8	0.5	*35.3*				15.2	3.4	989.0	425.0	*17.1*	4.3	1.7	Portable CReSS	day 3
		own brand (~6.5 mg tar)	20	♂/♀	30.9	12.5	17.3	6.6	56.0	14.4	1.6	0.4	*34.6*				14.2	3.4	847.0	292.0	*14.7*	4.3	1.7	Portable CReSS	day 5
Watson *et al* [72]	2019	own brand	163	♂/♀	42.4	18-66	16.1	6-40	54.1	CI 51.5-56.7	1.5	CI 1.48-1.59	*35.4*		16.8	CI 15.2-18.5	11.0	CI 9.8-12.1	*595.1*		*9.6*			CReSS	day 1
		own brand	163	♂/♀	42.4	18-66	16.1	6-40	53.6	CI 51.1-56.1	1.5	CI 1.47-1.58	*35.3*		16.3	CI 14.7-17.9	14.9	CI 13.4-16.5	*798.6*		*12.9*			CReSS	day 2

Calculated values in *italic*. SD = standard deviation, CI = 95% Confidence interval, SEM = Standard error of the mean, SE = Standard error, ♀ female, ♂ male, CRSSPT = Clinical Research Support System, Plowshare Technologies, CReSS = CReSSmicro™ (Clinical Research Support System), Plowshare Technologies. Blue: highest value. Yellow: lowest value. $ Data originates from ISO/TR 17219:2013 (stated as personal communication) [73]

References

1. Buchhalter, A.R.; Eissenberg, T. Preliminary evaluation of a novel smoking system: effects on subjective and physiological measures and on smoking behavior. Nicotine Tob Res 2000, 2, 39-43, doi:10.1080/14622200050011286.
2. Djordjevic, M.V.; Stellman, S.D.; Zang, E. Doses of nicotine and lung carcinogens delivered to cigarette smokers. J. Natl. Cancer Inst. 2000, 92, 106-111, doi:10.1093/jnci/92.2.106.
3. Breland, A.B.; Buchhalter, A.R.; Evans, S.E.; Eissenberg, T. Evaluating acute effects of potential reduced-exposure products for smokers: clinical laboratory methodology. Nicotine Tob Res 2002, 4 Suppl 2, S131-140, doi:10.1080/1462220021000032780.
4. Breland, A.B.; Evans, S.E.; Buchhalter, A.R.; Eissenberg, T. Acute effects of Advance: a potential reduced exposure product for smokers. Tob. Control 2002, 11, 376-378, doi:10.1136/tc.11.4.376.
5. Dixon, M.; Kochhar, N.; Prasad, K.; Shepperd, J.; Warburton, D.M. The influence of changing nicotine to tar ratios on human puffing behaviour and perceived sensory response. Psychopharmacology 2003, 170, 434-442, doi:10.1007/s00213-003-1541-8.
6. Lee, E.M.; Malson, J.L.; Waters, A.J.; Moolchan, E.T.; Pickworth, W.B. Smoking topography: reliability and validity in dependent smokers. Nicotine Tob Res 2003, 5, 673-679, doi:10.1080/1462220031000158645.
7. Aung, A.T.; Pickworth, W.B.; Moolchan, E.T. History of marijuana use and tobacco smoking topography in tobacco-dependent adolescents. Addict. Behav. 2004, 29, 699-706, doi:10.1016/j.addbeh.2004.02.012.
8. Hughes, J.R.; Hecht, S.S.; Carmella, S.G.; Murphy, S.E.; Callas, P. Smoking behaviour and toxin exposure during six weeks use of a potential reduced exposure product: Omni. Tob. Control 2004, 13, 175-179, doi:10.1136/tc.2003.005439.
9. Lee, E.M.; Malson, J.L.; Moolchan, E.T.; Pickworth, W.B. Quantitative comparisons between a nicotine delivery device (Eclipse) and conventional cigarette smoking. Nicotine Tob Res 2004, 6, 95-102, doi:10.1080/14622200310001656911.
10. Strasser, A.A.; Pickworth, W.B.; Patterson, F.; Lerman, C. Smoking topography predicts abstinence following treatment with nicotine replacement therapy. Cancer Epidemiol. Biomarkers Prev. 2004, 13, 1800-1804.
11. Wood, T.; Wewers, M.E.; Groner, J.; Ahijevych, K. Smoke constituent exposure and smoking topography of adolescent daily cigarette smokers. Nicotine Tob Res 2004, 6, 853-862, doi:10.1080/1462220042000282537.
12. Zimmerman, D.M.; Sehnert, S.S.; Epstein, D.H.; Pickworth, W.B.; Robinson, M.L.; Moolchan, E.T. Smoking topography and trajectory of asthmatic adolescents requesting cessation treatment. Prev. Med. 2004, 39, 940-942, doi:10.1016/j.ypmed.2004.03.032.
13. Hammond, D.; Fong, G.T.; Cummings, K.M.; Hyland, A. Smoking topography, brand switching, and nicotine delivery: results from an in vivo study. Cancer Epidemiol. Biomarkers Prev. 2005, 14, 1370-1375, doi:10.1158/1055-9965.EPI-04-0498.
14. Hitsman, B.; Spring, B.; Wolf, W.; Pingitore, R.; Crayton, J.W.; Hedeker, D. Effects of acute tryptophan depletion on negative symptoms and smoking topography in nicotine-dependent schizophrenics and nonpsychiatric controls. Neuropsychopharmacology 2005, 30, 640-648, doi:10.1038/sj.npp.1300651.
15. Strasser, A.A.; Ashare, R.L.; Kozlowski, L.T.; Pickworth, W.B. The effect of filter vent blocking and smoking topography on carbon monoxide levels in smokers. Pharmacol. Biochem. Behav. 2005, 82, 320-329, doi:10.1016/j.pbb.2005.09.001.
16. Tidey, J.W.; Rohsenow, D.J.; Kaplan, G.B.; Swift, R.M. Cigarette smoking topography in smokers with schizophrenia and matched non-psychiatric controls. Drug Alcohol Depend. 2005, 80, 259-265, doi:10.1016/j.drugalcdep.2005.04.002.
17. Breland, A.B.; Kleykamp, B.A.; Eissenberg, T. Clinical laboratory evaluation of potential reduced exposure products for smokers. Nicotine Tob Res 2006, 8, 727-738, doi:10.1080/14622200600789585.
18. Franken, F.H.; Pickworth, W.B.; Epstein, D.H.; Moolchan, E.T. Smoking rates and topography predict adolescent smoking cessation following treatment with nicotine replacement therapy. Cancer Epidemiol. Biomarkers Prev. 2006, 15, 154-157, doi:10.1158/1055-9965.EPI-05-0167.
19. Hammond, D.; Fong, G.T.; Cummings, K.M.; O'Connor, R.J.; Giovino, G.A.; McNeill, A. Cigarette yields and human exposure: a comparison of alternative testing regimens. Cancer Epidemiol. Biomarkers Prev. 2006, 15, 1495-1501, doi:10.1158/1055-9965.EPI-06-0047.
20. Kassel, J.D.; Greenstein, J.E.; Evatt, D.P.; Wardle, M.C.; Yates, M.C.; Veilleux, J.C.; Eissenberg, T. Smoking topography in response to denicotinized and high-yield nicotine cigarettes in adolescent smokers. J. Adolesc. Health 2007, 40, 54-60, doi:10.1016/j.jadohealth.2006.08.006.
21. Melikian, A.A.; Djordjevic, M.V.; Hosey, J.; Zhang, J.; Chen, S.; Zang, E.; Muscat, J.; Stellman, S.D. Gender differences relative to smoking behavior and emissions of toxins from mainstream cigarette smoke. Nicotine Tob Res 2007, 9, 377-387, doi:10.1080/14622200701188836.
22. Djordjevic, M.V.; Fan, J.; Ferguson, S.; Hoffmann, D. Self-regulation of smoking intensity. Smoke yields of the low-nicotine, low-'tar' cigarettes. Carcinogenesis 1995, 16, 2015-2021, doi:10.1093/carcin/16.9.2015.
23. Strasser, A.A.; Lerman, C.; Sanborn, P.M.; Pickworth, W.B.; Feldman, E.A. New lower nicotine cigarettes can produce compensatory smoking and increased carbon monoxide exposure. Drug Alcohol Depend. 2007, 86, 294-300, doi:10.1016/j.drugalcdep.2006.06.017.

24. Strasser, A.A.; Malaiyandi, V.; Hoffmann, E.; Tyndale, R.F.; Lerman, C. An association of CYP2A6 genotype and smoking topography. Nicotine Tob Res 2007, 9, 511-518, doi:10.1080/14622200701239605.

25. Attwood, A.S.; O'Sullivan, H.; Leonards, U.; Mackintosh, B.; Munafo, M.R. Attentional bias training and cue reactivity in cigarette smokers. Addiction 2008, 103, 1875-1882, doi:10.1111/j.1360-0443.2008.02335.x.

26. Collins, C.C.; Lippmann, B.M.; Lo, S.J.; Moolchan, E.T. Time spent with smoking parents and smoking topography in adolescents. Addict. Behav. 2008, 33, 1594-1597, doi:10.1016/j.addbeh.2008.07.003.

27. Rees, V.W.; Wayne, G.F.; Connolly, G.N. Puffing style and human exposure minimally altered by switching to a carbon-filtered cigarette. Cancer Epidemiol. Biomarkers Prev. 2008, 17, 2995-3003, doi:10.1158/1055-9965.EPI-07-2533.

28. Grainge, M.J.; Shahab, L.; Hammond, D.; O'Connor, R.J.; McNeill, A. First cigarette on waking and time of day as predictors of puffing behaviour in UK adult smokers. Drug Alcohol Depend. 2009, 101, 191-195, doi:10.1016/j.drugalcdep.2009.01.013.

29. Blank, M.D.; Disharoon, S.; Eissenberg, T. Comparison of methods for measurement of smoking behavior: mouthpiece-based computerized devices versus direct observation. Nicotine Tob Res 2009, 11, 896-903, doi:10.1093/ntr/ntp083.

30. Dickens, C.; McGrath, C.; Warren, N.; Biggs, P.; McAughey, J. Puffing and inhalation behaviour in cigarette smoking: Implications for particle diameter and dose. Journal of Physics: Conference Series 2009, 151, 012019.

31. Moolchan, E.T.; Parzynski, C.S.; Jaszyna-Gasior, M.; Collins, C.C.; Leff, M.K.; Zimmerman, D.L. A link between adolescent nicotine metabolism and smoking topography. Cancer Epidemiol. Biomarkers Prev. 2009, 18, 1578-1583, doi:10.1158/1055-9965.EPI-08-0592.

32. Collins, C.C.; Epstein, D.H.; Parzynski, C.S.; Zimmerman, D.; Moolchan, E.T.; Heishman, S.J. Puffing behavior during the smoking of a single cigarette in tobacco-dependent adolescents. Nicotine Tob Res 2010, 12, 164-167, doi:10.1093/ntr/ntp176.

33. O'Connor, R.J.; Rees, V.W.; Norton, K.J.; Cummings, K.M.; Connolly, G.N.; Alpert, H.R.; Sjodin, A.; Romanoff, L.; Li, Z.; June, K.M., et al. Does switching to reduced ignition propensity cigarettes alter smoking behavior or exposure to tobacco smoke constituents? Nicotine Tob Res 2010, 12, 1011-1018, doi:10.1093/ntr/ntq139.

34. June, K.M.; Norton, K.J.; Rees, V.W.; O'Connor, R.J. Influence of measurement setting and home smoking policy on smoking topography. Addict. Behav. 2012, 37, 42-46, doi:10.1016/j.addbeh.2011.07.039.

35. June, K.M.; Hammond, D.; Sjodin, A.; Li, Z.; Romanoff, L.; O'Connor, R.J. Cigarette ignition propensity, smoking behavior, and toxicant exposure: A natural experiment in Canada. Tob. Induc. Dis. 2011, 9, 13, doi:10.1186/1617-9625-9-13.

36. McKee, S.A.; Sinha, R.; Weinberger, A.H.; Sofuoglu, M.; Harrison, E.L.; Lavery, M.; Wanzer, J. Stress decreases the ability to resist smoking and potentiates smoking intensity and reward. J Psychopharmacol 2011, 25, 490-502, doi:10.1177/0269881110376694.

37. Strasser, A.A.; Benowitz, N.L.; Pinto, A.G.; Tang, K.Z.; Hecht, S.S.; Carmella, S.G.; Tyndale, R.F.; Lerman, C.E. Nicotine metabolite ratio predicts smoking topography and carcinogen biomarker level. Cancer Epidemiol. Biomarkers Prev. 2011, 20, 234-238, doi:10.1158/1055-9965.EPI-10-0674.

38. Veilleux, J.C.; Kassel, J.D.; Heinz, A.J.; Braun, A.; Wardle, M.C.; Greenstein, J.; Evatt, D.P.; Conrad, M. Predictors and sequelae of smoking topography over the course of a single cigarette in adolescent light smokers. J. Adolesc. Health 2011, 48, 176-181, doi:10.1016/j.jadohealth.2010.06.015.

39. Williams, J.M.; Gandhi, K.K.; Lu, S.E.; Kumar, S.; Steinberg, M.L.; Cottler, B.; Benowitz, N.L. Shorter interpuff interval is associated with higher nicotine intake in smokers with schizophrenia. Drug Alcohol Depend. 2011, 118, 313-319, doi:10.1016/j.drugalcdep.2011.04.009.

40. Williams, J.M.; Gandhi, K.K.; Lu, S.E.; Steinberg, M.L.; Benowitz, N.L. Nicotine intake and smoking topography in smokers with bipolar disorder. Bipolar Disord 2012, 14, 618-627, doi:10.1111/j.1399-5618.2012.01047.x.

41. Brinkman, M.C.; Chuang, J.C.; Gordon, S.M.; Kim, H.; Kroeger, R.R.; Polzin, G.M.; Richter, P.A. Exposure to and deposition of fine and ultrafine particles in smokers of menthol and nonmenthol cigarettes. Inhal. Toxicol. 2012, 24, 255-269, doi:10.3109/08958378.2012.667218.

42. Faulkner, G.E.; Arbour-Nicitopoulos, K.P.; Hsin, A. Cutting Down One Puff at a Time: The Acute Effects of Exercise on Smoking Behaviour. Journal of Smoking Cessation 2012, 5, 130-135, doi:10.1375/jsc.5.2.130.

43. Perkins, K.A.; Karelitz, J.L.; Giedgowd, G.E.; Conklin, C.A. The reliability of puff topography and subjective responses during ad lib smoking of a single cigarette. Nicotine Tob Res 2012, 14, 490-494, doi:10.1093/ntr/ntr150.

44. Matsumoto, M.; Inaba, Y.; Yamaguchi, I.; Endo, O.; Hammond, D.; Uchiyama, S.; Suzuki, G. Smoking topography and biomarkers of exposure among Japanese smokers: associations with cigarette emissions obtained using machine smoking protocols. Environ. Health Prev. Med. 2013, 18, 95-103, doi:10.1007/s12199-012-0293-7.

45. McClure, E.A.; Saladin, M.E.; Baker, N.L.; Carpenter, M.J.; Gray, K.M. Smoking topography and abstinence in adult female smokers. Addict. Behav. 2013, 38, 2833-2836, doi:10.1016/j.addbeh.2013.08.004.

46. Strasser, A.A.; Ashare, R.L.; Kaufman, M.; Tang, K.Z.; Mesaros, A.C.; Blair, I.A. The effect of menthol on cigarette smoking behaviors, biomarkers and subjective responses. Cancer Epidemiol. Biomarkers Prev. 2013, 22, 382-389, doi:10.1158/1055-9965.EPI-12-1097.

47. Tidey, J.W.; Rohsenow, D.J.; Kaplan, G.B.; Swift, R.M.; Ahnallen, C.G. Separate and combined effects of very low nicotine cigarettes and nicotine replacement in smokers with schizophrenia and controls. Nicotine Tob Res 2013, 15, 121-129, doi:10.1093/ntr/nts098.
48. Hammond, D.; O'Connor, R.J. Reduced nicotine cigarettes: smoking behavior and biomarkers of exposure among smokers not intending to quit. Cancer Epidemiol. Biomarkers Prev. 2014, 23, 2032-2040, doi:10.1158/1055-9965.Epi-13-0957.
49. Norton, K.J.; June, K.M.; O'Connor, R.J. Initial puffing behaviors and subjective responses differ between an electronic nicotine delivery system and traditional cigarettes. Tob. Induc. Dis. 2014, 12, 17, doi:10.1186/1617-9625-12-17.
50. Schneider, T.; De Jesus, S.; Prapavessis, H. The Effect of Acute Exercise on Smoking Topography: No Evidence for Cutting Down One Puff at a Time. Journal of Smoking Cessation 2014, 10, 146-153, doi:10.1017/jsc.2014.2.
51. Appleton, S.; Liu, J.; Lipowicz, P.J.; Sarkar, M. Effect of cigarette design on biomarkers of exposure, puffing topography and respiratory parameters. Inhal. Toxicol. 2015, 27, 174-180, doi:10.3109/08958378.2015.1021980.
52. Brinkman, M.C.; Kim, H.; Chuang, J.C.; Kroeger, R.R.; Deojay, D.; Clark, P.I.; Gordon, S.M. Comparison of True and Smoothed Puff Profile Replication on Smoking Behavior and Mainstream Smoke Emissions. Chem. Res. Toxicol. 2015, 28, 182-190, doi:10.1021/tx500318h.
53. Chung, S.; Kim, S.S.; Kini, N.; Fang, H.J.; Kalman, D.; Ziedonis, D.M. Smoking topography in Korean American and white men: preliminary findings. J Immigr Minor Health 2015, 17, 860-866, doi:10.1007/s10903-013-9921-6.
54. Gass, J.C.; Germeroth, L.J.; Wray, J.M.; Tiffany, S.T. The Reliability and Stability of Puff Topography Variables in Non-Daily Smokers Assessed in the Laboratory. Nicotine Tob Res 2016, 18, 484-490, doi:10.1093/ntr/ntv045.
55. Krebs, N.M.; Chen, A.; Zhu, J.; Sun, D.; Liao, J.; Stennett, A.L.; Muscat, J.E. Comparison of Puff Volume With Cigarettes per Day in Predicting Nicotine Uptake Among Daily Smokers. Am. J. Epidemiol. 2016, 184, 48-57, doi:10.1093/aje/kwv341.
56. Chen, A.; Krebs, N.M.; Zhu, J.; Muscat, J.E. Nicotine metabolite ratio predicts smoking topography: The Pennsylvania Adult Smoking Study. Drug Alcohol Depend. 2018, 190, 89-93, doi:10.1016/j.drugalcdep.2018.06.003.
57. Mercincavage, M.; Souprountchouk, V.; Tang, K.Z.; Dumont, R.L.; Wileyto, E.P.; Carmella, S.G.; Hecht, S.S.; Strasser, A.A. A Randomized Controlled Trial of Progressively Reduced Nicotine Content Cigarettes on Smoking Behaviors, Biomarkers of Exposure, and Subjective Ratings. Cancer Epidemiol. Biomarkers Prev. 2016, 25, 1125-1133, doi:10.1158/1055-9965.EPI-15-1088.
58. Mercincavage, M.; Lochbuehler, K.; Wileyto, E.P.; Benowitz, N.L.; Tyndale, R.F.; Lerman, C.; Strasser, A.A. Association of Reduced Nicotine Content Cigarettes With Smoking Behaviors and Biomarkers of Exposure Among Slow and Fast Nicotine Metabolizers: A Nonrandomized Clinical Trial. JAMA Netw Open 2018, 1, e181346-e181346, doi:10.1001/jamanetworkopen.2018.1346.
59. Pulcu, E. Self-report distortions of puffing topography in daily smokers. J. Health Psychol. 2016, 21, 1644-1654, doi:10.1177/1359105314560917.
60. Ross, K.C.; Dempsey, D.A.; St Helen, G.; Delucchi, K.; Benowitz, N.L. The Influence of Puff Characteristics, Nicotine Dependence, and Rate of Nicotine Metabolism on Daily Nicotine Exposure in African American Smokers. Cancer Epidemiol. Biomarkers Prev. 2016, 25, 936-943, doi:10.1158/1055-9965.EPI-15-1034.
61. Ross, K.C.; Juliano, L.M. Smoking Through a Topography Device Diminishes Some of the Acute Rewarding Effects of Smoking. Nicotine Tob Res 2016, 18, 564-571, doi:10.1093/ntr/ntv159.
62. Tidey, J.W.; Cassidy, R.N.; Miller, M.E. Smoking Topography Characteristics of Very Low Nicotine Content Cigarettes, With and Without Nicotine Replacement, in Smokers With Schizophrenia and Controls. Nicotine Tob Res 2016, 18, 1807-1812, doi:10.1093/ntr/ntw089.
63. Farris, S.G.; Aston, E.R.; Abrantes, A.M.; Zvolensky, M.J. Tobacco demand, delay discounting, and smoking topography among smokers with and without psychopathology. Drug Alcohol Depend. 2017, 179, 247-253, doi:10.1016/j.drugalcdep.2017.06.042.
64. Higgins, S.T.; Heil, S.H.; Sigmon, S.C.; Tidey, J.W.; Gaalema, D.E.; Stitzer, M.L.; Durand, H.; Bunn, J.Y.; Priest, J.S.; Arger, C.A., et al. Response to varying the nicotine content of cigarettes in vulnerable populations: an initial experimental examination of acute effects. Psychopharmacology 2017, 234, 89-98, doi:10.1007/s00213-016-4438-z.
65. Hsu, P.C.; Lan, R.S.; Brasky, T.M.; Marian, C.; Cheema, A.K.; Ressom, H.W.; Loffredo, C.A.; Pickworth, W.B.; Shields, P.G. Menthol Smokers: Metabolomic Profiling and Smoking Behavior. Cancer Epidemiol. Biomarkers Prev. 2017, 26, 51-60, doi:10.1158/1055-9965.EPI-16-0124.
66. Watson, C.V.; Richter, P.; de Castro, B.R.; Sosnoff, C.; Potts, J.; Clark, P.; McCraw, J.; Yan, X.; Chambers, D.; Watson, C. Smoking Behavior and Exposure: Results of a Menthol Cigarette Cross-over Study. Am. J. Health Behav. 2017, 41, 309-319, doi:10.5993/AJHB.41.3.10.
67. Bergeria, C.L.; Heil, S.H.; Bunn, J.Y.; Sigmon, S.C.; Higgins, S.T. Comparing Smoking Topography and Subjective Measures of Usual Brand Cigarettes Between Pregnant and Non-Pregnant Smokers. Nicotine Tob Res 2018, 20, 1243-1249, doi:10.1093/ntr/ntx148.

68. Kim, S.; Yu, S. Smoking Topography among Korean Smokers: Intensive Smoking Behavior with Larger Puff Volume and Shorter Interpuff Interval. Int. J. Environ. Res. Public Health 2018, 15, doi:10.3390/ijerph15051024.
69. Shiffman, S.; Scholl, S. Increases in Cigarette Consumption and Decreases in Smoking Intensity When Nondaily Smokers Are Provided With Free Cigarettes. Nicotine Tob Res 2018, 20, 1237-1242, doi:10.1093/ntr/ntx221.
70. Vansickel, A.R.; Edmiston, J.S.; Liang, Q.; Duhon, C.; Connell, C.; Bennett, D.; Sarkar, M. Characterization of puff topography of a prototype electronic cigarette in adult exclusive cigarette smokers and adult exclusive electronic cigarette users. Regul. Toxicol. Pharmacol. 2018, 98, 250-256, doi:10.1016/j.yrtph.2018.07.019.
71. Yuki, D.; Takeshige, Y.; Nakaya, K.; Futamura, Y. Assessment of the exposure to harmful and potentially harmful constituents in healthy Japanese smokers using a novel tobacco vapor product compared with conventional cigarettes and smoking abstinence. Regul. Toxicol. Pharmacol. 2018, 96, 127-134, doi:10.1016/j.yrtph.2018.05.001.
72. Watson, C.V.; Richter, P.; Li, Y.; Phillips, T.; Pickworth, W.B.; deCastro, R.B.; Potts, J.; Watson, C. Mouth Level Nicotine in a Clinical Setting versus Non-clinical Setting. Tobacco Regulatory Science 2019, 5, 229-241, doi:10.18001/trs.5.3.2.
73. ISO/TR 17219:2013 Review of human smoking behaviour and recommendations for a new ISO standard for the machine smoking of cigarettes; International Organisation for Standardisation: Geneva, Switzerland, 2013.
